# Large-scale copy number alterations are enriched for synthetic viability in BRCA1/BRCA2 tumors

**DOI:** 10.1186/s13073-024-01371-y

**Published:** 2024-08-28

**Authors:** Yingjie Zhu, Xin Pei, Ardijana Novaj, Jeremy Setton, Daniel Bronder, Fatemeh Derakhshan, Pier Selenica, Niamh McDermott, Mehmet Orman, Sarina Plum, Shyamal Subramanyan, Sara H. Braverman, Biko McMillan, Sonali Sinha, Jennifer Ma, Andrea Gazzo, Atif Khan, Samuel Bakhoum, Simon N. Powell, Jorge S. Reis-Filho, Nadeem Riaz

**Affiliations:** 1 Department of Pathology and Laboratory Medicine, Memorial Sloan Kettering Cancer Center, New York, NY USA; 2 Department of Radiation Oncology, Memorial Sloan Kettering Cancer Center, New York, NY USA; 3 Human Oncology and Pathogenesis Program, Memorial Sloan Kettering Cancer Center, New York, NY USA; 4 Present address: Department of Pathology & Cell Biology, Columbia University Medical Center, New York, NY USA

**Keywords:** BRCA1, BRCA2, Copy number alterations, Gene expression, Synthetic viability, CRISPR-Cas9 knockout, TCGA, ICGC, DNA repair

## Abstract

**Background:**

Pathogenic *BRCA1* or *BRCA2* germline mutations contribute to hereditary breast, ovarian, prostate, and pancreatic cancer. Paradoxically, bi-allelic inactivation of *BRCA1* or *BRCA2* (bBRCA1/2) is embryonically lethal and decreases cellular proliferation*.* The compensatory mechanisms that facilitate oncogenesis in bBRCA1/2 tumors remain unclear.

**Methods:**

We identified recurrent genetic alterations enriched in human bBRCA1/2 tumors and experimentally validated if these improved proliferation in cellular models. We analyzed mutations and copy number alterations (CNAs) in bBRCA1/2 breast and ovarian cancer from the TCGA and ICGC. We used Fisher’s exact test to identify CNAs enriched in bBRCA1/2 tumors compared to control tumors that lacked evidence of homologous recombination deficiency. Genes located in CNA regions enriched in bBRCA1/2 tumors were further screened by gene expression and their effects on proliferation in genome-wide CRISPR/Cas9 screens. A set of candidate genes was functionally validated with in vitro clonogenic survival and functional assays to validate their influence on proliferation in the setting of bBRCA1/2 mutations.

**Results:**

We found that bBRCA1/2 tumors harbor recurrent large-scale genomic deletions significantly more frequently than histologically matched controls (*n* = 238 cytobands in breast and ovarian cancers). Within the deleted regions, we identified 277 BRCA1-related genes and 218 BRCA2-related genes that had reduced expression and increased proliferation in bBRCA1/2 but not in wild-type cells in genome-wide CRISPR screens. In vitro validation of 20 candidate genes with clonogenic proliferation assays validated 9 genes, including *RIC8A* and *ATMIN* (ATM-Interacting protein). We identified loss of *RIC8A*, which occurs frequently in both bBRCA1/2 tumors and is synthetically viable with loss of both *BRCA1* and *BRCA2*. Furthermore, we found that metastatic homologous recombination deficient cancers acquire loss-of-function mutations in *RIC8A*. Lastly, we identified that *RIC8A* does not rescue homologous recombination deficiency but may influence mitosis in bBRCA1/2 tumors, potentially leading to increased micronuclei formation.

**Conclusions:**

This study provides a means to solve the tumor suppressor paradox by identifying synthetic viability interactions and causal driver genes affected by large-scale CNAs in human cancers.

**Supplementary Information:**

The online version contains supplementary material available at 10.1186/s13073-024-01371-y.

## Background

Germline mutations in BRCA1/BRCA2 lead to a hereditary cancer predisposition syndrome that can increase the risk of ovarian, breast, prostate, and pancreatic cancer, among others [1–3]. Up to 40–80% of hereditary breast and ovarian cancers are due to BRCA1/BRCA2 germline mutations [4], and these genes are affected by germline or somatic genetic alterations in up to 8% of cancers. Bi-allelic inactivation of *BRCA1* or *BRCA2* (bBRCA1/2) results in homologous recombination (HR) DNA repair deficiency (HRD), resulting in genomic instability with well-described mutational signatures [5, 6]. Thus, BRCA1 and BRCA2 are recognized as tumor suppressors, and their loss serves as an enabling hallmark of cancer by increasing genomic instability. This, in turn, facilitates the acquisition of additional alterations necessary for oncogenesis.

Unlike many other tumor suppressors, BRCA1/BRCA2 inactivation leads to embryonic lethality in mice [7, 8] and inhibits cellular proliferation in human and murine cells in vitro [9]. Furthermore, recent pan-genome knockout screens in human and murine lines often identify BRCA1/BRCA2 as an essential gene that significantly reduces proliferation (Additional file 1: Fig. S1) [10, 11]. Consequently, despite their role in causing early-onset breast and ovarian tumors when inactivated, the loss of these genes surprisingly impedes cellular viability, a function vital for cancer development. One conceptual resolution for these paradoxical observations is the development of synthetic viability during oncogenesis. Unlike synthetic lethality, synthetic viability entails the rescue of lethal effects resulting from a mutation in one gene by a co-occurring alteration in a second gene [12]. A well-described instance of a synthetic viable interaction involves BRCA1 and 53BP1. In murine models, the loss of 53BP1, in part rescues BRCA1 deficiency; however, this interaction is not commonly observed in human cancers [13, 14]. Similarly, hypomorphic *BRCA1* mouse mutants can be rescued from embryonic lethality by *TP53* mutation [15–18]. In the human context, mutations in *TP53* frequently occur in bBRCA1/2 tumors; however, they are known to only modestly reverse the decreased proliferative phenotype [19, 20].

Several studies have investigated whether an enrichment for mutations affecting other genes would be detected in BRCA1 or BRCA2 cancers and provide a basis for the synthetic viable interactions. These studies, however, revealed minimal distinctions when comparing BRCA1- or BRCA2-related breast cancers, matched for age and subtype, with sporadic controls [19, 21, 22]. While *BRCA1*/*BRCA2* mutations modestly increase tumor mutation burden, their impact on the copy number landscape of cancer is notably more substantial [23]. Prior studies [24, 25] have demonstrated the significance of large-scale copy number alterations in tumor development by altering levels of tumor suppressors and oncogenes. These analyses highlighted that tumor suppressors, which inhibit cell proliferation, tend to be enriched in recurring focal deletions, while oncogenes, which promote proliferation, are often found in amplifications. The considerable size of recurrently altered chromosomal regions has posed challenges for the use of computational approaches to pinpoint specific genes that would be causally linked to oncogenesis in this context [26].

**Fig. 1:**
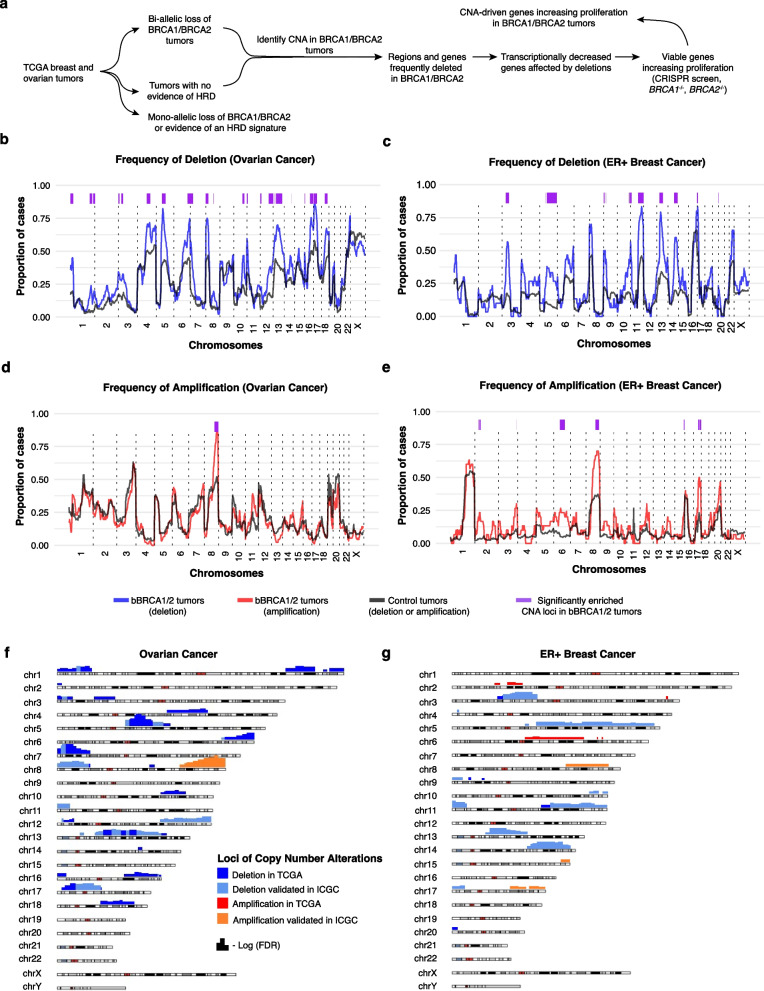
The repertoire of copy number alterations in BRCA1/BRCA2-mutated tumors. **a** A scheme figure of computational analysis to identify copy number altered genes that may increase proliferation of BRCA1/BRCA2 tumors. Tumors from TCGA were divided into three categories: (1) tumors harboring bBRCA1/2 mutations; (2) non-bBRCA1/2 mutated tumors with high levels of mutational signature 3 exposure and large-scale transition (LST); (3) wild-type tumors with low mutational signature 3 exposure and LST (see the “Methods” section). The subsequent analysis sought to evaluate genetic alterations enriched in bBRCA1/2 tumors compared to wild-type tumors. Transcriptionally decreased genes within copy number deleted regions were identified and intersected with viable genes synthetically promoting cell proliferation of *BRCA1*^−/−^ and *BRCA2*^−/−^ tumors. **b**, **c** Genome-wide analysis of copy number deletion in bBRCA1/2 tumors compared to controls in OV (**b**) and ER+ BC (**c**). Amplifications were defined by an absolute copy number of at least the average tumor ploidy + 3. Deletions were defined by an absolute copy number less than the ploidy of the tumor. Significant loci defined by the two-sided Fisher’s exact test were highlighted in purple color. **d**, **e** are same as **b** and **c**, but for copy number amplification. bBRCA1/2 OV tumors (*n* = 89); control OV tumors (*n* = 136); bBRCA1/2 ER+ tumors (*n* = 29); control ER+ tumors (*n* = 420). **f** Chromosomal ideogram for enriched copy number segments in bBRCA1/2 OV. Cytobands enriched for copy number deletion in TCGA are colored in blue (FDR < 0.05) and cyan for those regions validated in ICGC. For cytobands enriched for copy number amplification, red demarcates cytobands identified in TCGA (FDR < 0.05) and orange demarcates cytobands validated in ICGC. **g** Chromosomal ideogram for ER+ BC using the same color scheme and conventions as in **f**

Here, we hypothesize that the necessary genetic modifications for oncogenesis in the context of *BRCA1*/*BRCA2* inactivation may arise from recurrent copy number alterations (CNAs) present in these neoplasms. We posit that inactivation of certain genes through copy number deletion can promote cellular viability in the context of *BRCA1*/*BRCA2* deficiency and lead to synthetic viability. Consequently, we conducted a comprehensive assessment of cancer genomic data to identify co-occurring genetic alterations in BRCA1/BRCA2 cancers, comparing them with wild-type histologic controls, to identify candidate genes that would be in a synthetic viable interaction with BRCA1 or BRCA2 loss-of-function (Fig. 1a). By leveraging genome-wide CRISPR screens, we narrowed down the synthetic viable candidates that were identified from cancer genetic data and experimentally validated several genes associated with synthetic viability. Among several novel synthetic viable interactions discovered, we have identified loss of *RIC8A*, a recently identified recurrently mutated gene in metastatic breast cancer, as being synthetically viable with both BRCA1 and BRCA2 loss-of-function. Taken together, here we have identified new synthetic viable interactions for bBRCA1/2 malignancies and illustrated a high-throughput framework for the identification of genes whose large-scale CNAs facilitate oncogenesis.

## Methods

### Cohorts of bi-allelic BRCA1/BRCA2 patients

For the identification of recurrently amplified and deleted genomic loci, breast and ovarian cancers from The Cancer Genome Atlas (TCGA) [27, 28] served as the discovery cohort. Data from the International Cancer Genome Consortium (ICGC)/Pan-Cancer Analysis of Whole Genomes working group (PCAWG) (noted as ICGC below for consistency) [29] for breast and ovarian cancer patients served as the validation cohort. For the TCGA cohort, whole-exome sequencing data from ovarian cancer (OV, *n* = 417), ER+ breast cancer (ER+ BC, *n* = 581), or triple-negative breast cancer (TNBC, *n* = 143) was used to identify mutations, copy number alterations, and SBS mutational signatures. Mutational data were obtained from the published TCGA MC3 Public MAF dataset [28]. Bi-allelic *BRCA1*/*BRCA2* status and mutational signatures were obtained in a similar manner as we had previously published [5] (Additional file 2: Table S1). Copy number analysis and identification of tumor ploidy were performed using FACETS [30]. For the ovarian cancer validation cohort from ICGC (*n* = 69), somatic mutations and germline mutations were obtained from the ICGC data portal and annotated with VEP [31] package. Bi-allelic mutational status was determined as described in our previous work [5]. For ICGC BC (ER+ , *n* = 320; TNBC, *n* = 162), somatic, germline mutations, and bi-allelic BRCA1/BRCA2 status were obtained from previous publications [6]. For the TCGA cohort, RNA-seq data from OV (36 bBRCA1 and 85 controls) and ER+ BC (20 bBRCA and 421 controls) were used to identify transcriptionally decreased genes.

### Identification of recurrent genetic alterations in bi-allelic BRCA1/BRCA2 tumors

Copy number data for each individual tumor was obtained to assign each of 851 cytobands (excluding chromosome Y) an integer copy number by identifying copy number segments that overlapped with the cytoband (> 50% overlap) using BEDTools [32]. We defined a cytoband as deleted when its copy number was less than or equal to (tumor ploidy − 1). Copy number amplifications were identified when a cytoband copy number was larger than or equal to (tumor ploidy + 3). We evaluated whether a segment was deleted or amplified at a higher frequency in bi-allelic BRCA1/BRCA2 tumors than control tumors with Fisher’s test and corrected for multiple testing by controlling the false discovery rate using the method of Benjamini and Hochberg (FDR < 0.05). BRCA1/BRCA2 wild-type tumors were those tumors without a mono-allelic alteration in BRCA1/BRCA2 and little evidence of mutational signatures of HRD features (non-dominant signature 3 and LST < 12).

To ensure that enriched cytobands were not solely due to high levels of genomic instability in bi-allelic BRCA1/BRCA2 tumors, we performed permutation testing controlling sample level and cytoband level instability, similar to previous testing we have performed [33, 34] through EcoSimR package [35]. Briefly, in this test, we permutated the cytoband copy number matrix (cytoband x samples, with copy number change coded by 0 or 1) by keeping the row and column sums constant to avoid other confounding factor group distributions. We performed this permutation 100,000 times and counted the number of significantly enriched cytobands in each iteration, therefore calculating an empirical *P*-value for the observed significantly enriched cytoband numbers in the bi-allelic BRCA1/BRCA2 samples.

Co-occurring somatic mutations with bi-allelic BRCA1/BRCA2 alterations were tested using Fisher’s exact test, first with recurrently mutated genes in each cancer type. We also evaluated 572 genes from COSMIC Cancer Gene Census [36]. Multiple testing was adjusted by using the false discovery rate correction with *q*-value < 0.10.

### Gene expression analysis

Both RNA-seq read counts and FPKM (Fragments Per Kilobase of transcript per Million mapped reads) values were obtained from TCGA and used for quality control and gene filtering. We first sought to identify differentially expressed genes between bBRCA1/BRCA2 and control tumors. We used RNA-Seq read counts and tested for differential expression based on a model using the negative binomial distribution embedded in DESeq2 [37].

To evaluate gene transcriptional consistency with copy number deletion, we defined a transcriptional consistency score (TCS). At first, gene expression levels for the tumors were categorized into high and low based on the median of FPKM values (all tumors regardless of their BRCA1/BRCA2 status or copy number status). For BRCA1/BRCA2 tumors, the samples were divided into two groups according to whether they were deleted or not. For the deleted tumors, TCS was calculated as the proportion of low-expression tumors. To address some genes that may have a small number of samples with recurrent deletion in BRCA1/BRCA2 tumors, only genes having at least 5 BRCA1/BRCA2 tumors with recurrent deletion were used to evaluate transcriptional consistency, that means, for example, 4 low expression tumors out of 5 can give TCS as 0.8. Moreover, we normalized TCS for the BRCA1/BRCA2 tumors by adjusting to the value of the TCS obtained in control tumors without the locus deleted. Adjusting TCS to not deleted-control tumors ensure decreased gene expression in BRCA1/BRCA2 tumors with recurrent deletion contributed by copy number deletion rather than other factors. Both TCS > 0.7 and normalized TCS > 1.5 (50% higher than control tumors) were used to identify genes with a consistent relationship between copy number deletion and decreased expression levels.

### De-convolution of **cancer**-specific gene expression

To identify genes transcriptionally affected by copy number from cancer cells rather than other non-cancer cells, we applied a de-convolution approach called BayesPrism, which uses bulk RNA-seq data and a reference single-cell expression data set to impute cancer-specific gene expression [38]. We obtained the original counts and annotated cell types of single-cell gene expression data in breast and ovary tissues from The Human Protein Atlas database [39]. We used raw counts of bulk RNA-seq data from TCGA as input. According to the BayesPrism tutorial, we excluded gene outliers expressed at high magnitudes, such as ribosomal protein genes and mitochondrial genes, which can dominate the distribution and bias the inference. At last, we extracted the posterior mean of the cancer-specific gene expression count matrix using glandular cells and myoepithelial cells in breast cancer and granulosa cells in ovarian cancer. The imputed counts were further converted into FPKM values for further analysis to identify transcriptionally consistent genes.

### CRISPR screen

A genome-wide CRISPR screen was performed using the human Brunello knockout (KO) library (targeting 19,114 genes with a total of 77,441 sgRNAs (4 sgRNAs per gene). Lentivirus carrying human CRISPR Brunello lentiviral pooled sgRNA library was produced in 293 T cells. DLD1 isogenic cells were transduced (0.3 MOI) with the lentiviral Brunello sgRNA library to maintain > 500 × gRNA representation. Following puromycin selection (1.5 μg/mL), surviving cells were allowed to proliferate for 14 days, with cell pellets harvested in triplicate at day 0 and day 14. Guide RNA cassettes were amplified from extracted genomic DNA to generate Illumina sequencing libraries. Namely, 3 μg of genomic DNA was added per 50 μl PCR reaction using staggered primers to increase base diversity. PCR products were then pooled and purified using QIAquick PCR purification kits (Qiagen). Pooled samples were sequenced by Illumina Novaseq. sgRNA read counts were first collected using MAGeCK count [40]. The sgRNA read counts were further analyzed using BAGEL algorithm (version 2) [41].

We noted that the cellular models (i.e., RPE1 and DLD1) used in the CRISPR screens in both Alvarez-Quilon et al. [42] and this work were TP53-mutated lines. The RPE1 cells were initially generated by Zimmermann et al. [43].

### Evaluating CRISPR screens of BRCA1/BRCA2 deficient cells to identify genes increasing cellular proliferation

To identify genes potentially increasing cell proliferation in *BRCA1*^−/−^ or *BRCA2*^−/−^ cells, we analyzed data on a BRCA1 knockout in RPE1 cells and BRCA2 knockout in DLD1 cells [42] using BAGEL algorithm (version 2) [41]. Bayes factor (BF) was calculated to quantify the degree of gene essentiality: a positive BF score represents a gene that is more likely to be essential, and a negative BF score identifies genes that are likely non-essential. To identify genes that were more likely to increase cell proliferation in *BRCA1*^−/−^ or *BRCA2*^−/−^ cells in comparison to wild-type cells, we introduced a relative proliferation score (RPS) to evaluate the relative proliferation in *BRCA1*^−/−^ or *BRCA2*^−/−^ cells compared with WT cells. The RPS was calculated by subtracting the BF score in WT cells from *BRCA1*^−/−^ or *BRCA2*^−/−^ cells, as shown below. The subtracted BF score, for example, BF_*BRCA1-/-*_ – BF_*WT*_, was comparable with fold change but considering the differences of posterior essential (*Pr(Ess-BRCA1*^−*/*−^*)*, *Pr(Ess-WT)*) probability and non-essential (*Pr(Non-BRCA1*^−*/*−^*)*, *Pr(Non-WT)*) probability [41, 44].

$$\begin{array}{c}Relative\ proliferation\ score{=BF}_{{BRCA1}^{-/-}}- {BF}_{WT}\\ =\text{log}\frac{Pr(Ess-{BRCA1}^{-/-})}{Pr(Non-{BRCA1}^{-/-})}-\text{log}\frac{Pr(Ess-WT)}{Pr(Non-WT)}\\ =\text{log}(\frac{\mathit{Pr}\left(Ess-{BRCA1}^{-/-}\right)}{\mathit{Pr}\left(Non-{BRCA1}^{-/-}\right)}/\frac{\mathit{Pr}\left(Ess-WT\right)}{\mathit{Pr}\left(Non-WT\right)})\end{array}$$

Similar to the BF score, RPS < 0 indicates relatively increased cell proliferation in *BRCA1*^−/−^ or *BRCA2*^−/−^ cells compared with WT cells, and RPS > 0 indicates relatively decreased cell proliferation. We considered the top 50% of the genes with negative RPS as increasing cell proliferation.

### DepMap gene effect analysis

To evaluate the synthetic viability of our BRCA1/BRCA2 candidates, we analyzed CRISPR data from the latest DepMap version 23Q4 [45]. We evaluated 1100 cell models in DepMap with CRISPR data, of which 55 loss-of-function (LoF) mutations were mutated in BRCA1 and/or BRCA2. However, most of these mutations are mono-allelic. Only the following lines are known to be bi-allelic in BRCA1 (*n* = 3): HCC1937_BREAST (ACH-000223), JHOS2_OVARY (ACH-000132), and HCC1395_BREAST (ACH-000699), and in BRCA2 (*n* = 1): CAPAN1_PANCREA (ACH-000354). Since genetic alterations in BRCA1 or BRCA2 are required to have both alleles knocked out to produce a phenotype. Hence, we focused our analysis on BRCA1, as multiple cell lines with bi-allelic alterations (*n* = 3 cell lines) were available for analysis. To define a threshold of gene effect and whether gene knock-out promotes cell proliferation, we examined known essential and non-essential genes provided by DepMap and considered gene effect values > -0.5 to promote proliferation.

### Functional enrichment analysis

Functional enrichment analysis was performed for the identified genes to find an overrepresentation of biological processes in Reactome [46] using gprofiler2 with gost function [47].

### Gene selection criteria for clonogenic assay

We first identified genes to validate for synthetic viability for BRCA1. We chose 9 genes from the top 50 candidates that conform to an essential to non-essential category switch (genes ranked according to relative proliferation score). These genes were selected based on their roles potentially promoting proliferation, their known involvement in DNA repair (e.g., ATMIN) [48] and/or diverse known cellular effects. For the gene essential to non-essential category switch, we classified genes into three categories in BRCA1/BRCA2 knockdown cells and WT cells separately according to the Bayes Factor scores: non-essential, neutral, and essential (where non-essential genes were defined as the bottom 50% genes with lowest BF score in BF score < 0, essential genes were defined as the top 50% genes with highest BF score in BF score > 0, and other genes are neutral). Genes promoting cell proliferation in BRCA1 and BRCA2 knockdown cells compared with WT cells were those categories switched from essential to neutral or non-essential, and from neutral to non-essential.

To identify candidates experimentally validating for BRCA2, we focused on candidates that were also identified for BRCA1 (i.e., *RIC8A*, *MMS19*, *NUP98*, *SUZ12*, *CDK7*, *CCNH*, *GNA12*) or were known tumor suppressors (*CDK12*, *NF1*, *EED*, *CCNK*).

### Clonogenic survival assay

Lentiviruses were created containing a CRISPR/Cas9 plasmid expressing GFP or BFP along with a guide for the gene of interest. Guides for each target gene are available in Additional file 3: Table S2. MCF12A cells were transduced with lentiviruses and cells expressing GFP or BFP were selected with FACS. Cells were transfected with either siBRCA1 (Horizon Discovery Ltd.—BRCA1- Catalog ID:L-003461–00-0005), siBRCA2 (BRCA2- Catalog ID:L-003462–00-0005), or siNT using Lipofectamine. Clonogenic cell survival assays were performed 48 h after siRNA transfection as previously described [49, 50]. Briefly, cells were plated for each condition in triplicate in 6-well plates and were fixed and counted for survival on day 8. Western blotting was performed to verify knockdown of BRCA1 (OP92, Sigma-Aldrich) and BRCA2 (OP95, Sigma-Aldrich).

### RIC8A cell viability assay in PARP inhibitor-treated cells

Two hundred cells of each genotype were seeded in a 96-well plate, with 4 technical replicates per dose, and 3 biological replicates performed. One hundred thirty-seven micrograms of olaparib was added on day 1 into treated wells and DMSO into control wells. Four days after plating, CellTiteGlo was used to evaluate viability and read for luminescence.

### RIC8A flowcytometry

Cells were collected by trypsinization and subsequently fixed in 70% ethanol and stored at – 20 °C overnight. Next, cells were permeabilized using 0.5% Triton-X-100 in PBS. Primary antibody against phospho-histone-H3 (1:1,000, Phospho-Histone H3 (Ser10) (D2C8) XP® Rabbit mAb #3377) was added in PBS containing 1% BSA, and samples were incubated overnight at 4 °C. Cells were washed, secondary antibody (1:500, Donkey anti-Rabbit IgG (H + L) Highly Cross-Adsorbed Secondary Antibody, Alexa Fluor Plus 647) was added, and samples were once again incubated overnight. After washing, 1 ml PBS containing 10 µg/ml DAPI was added per 10^6^ cells. All washes were performed with PBS. Analysis was performed on Cytek Biosciences Aurora flow cytometer.

### RIC8A immunofluorescence

Cells were grown on 19 mm coverslips in 12-well microtiter plates, fixed with ice-cold methanol, and permeabilized with 0.5% Triton-X-100 in PBS containing 1% BSA. Primary antibody against human centromere (1:1,000, Antibodies Incorporated, 15–234-0001) was added in in PBS containing 1% BSA, and samples were incubated overnight at 4 °C. Next, cells were washed, secondary antibody (1:500, Goat anti Human IgG (H + L) Secondary Antibody, Alexa Fluor 568, Invitrogen) was added, and samples were incubated overnight at 4 °C. Lastly, cells were counter-stained with DAPI (2 µg/ml DAPI in PBS) for 10 min at room temperature and mounted using Prolong Diamond Antifade Mountant (Life Technologies, P36961). Analysis was performed on Zeiss LSM 880 Airyscan.

### Analysis of RIC8A mutations

Clinical data, LST class (LST-high and LST-low), and SBS Signature 3 data were extracted from a cohort of metastatic breast cancer patients (*n* = 617) in the dataset published by Bertucci et al. [51] Among them, there were 410 ER+ breast cancer patients. LoF mutations in *RIC8A* were identified by integrating single nucleotide variants and frameshift truncation mutations. The term “BRCAness” refers to the phenotypic characteristics shared by BRCA1 and/or BRCA2 mutation carriers, predominantly reflecting homologous recombination deficiency. BRCAness tumors were defined by Bertucci et al. [51] Briefly, tumors displayed with high LST and more than 20% contribution from SBS Signature 3 were considered BRCAness.

### Statistics and reproducibility

The two-sided Fisher’s exact test was applied to identify enriched genomic loci with copy number alterations; *P*-values were further corrected for multiple comparisons using Benjamini and Hochberg method. In other analyses to test whether genes in deleted loci were enriched with transcriptionally decreased genes, tumor suppressors, and genes increasing proliferation, the two-sided Fisher’s exact test or hypergeometric test were applied as described in figure legends; error bars represent standard errors estimated using a proportion test. The measurements were taken from distinct samples. The plating efficiency of candidate genes with siBRCA1 or siBRCA2 was compared using the two-sided Student’s *t*-test in GraphPad Prism.

## Results

### Multiple chromosomal segment deletions are enriched in BRCA1/BRCA2 malignancies

We first sought to identify co-occurring mutated genes that were enriched in bBRCA1/2 tumors utilizing data from The Cancer Genome Atlas (TCGA), including ovarian cancer (OV, *n* = 417), ER+ breast cancer (ER+ BC, *n* = 581), or triple-negative breast cancer (TNBC, *n* = 143) cohorts (Additional file 1: Fig. S2a-c; Additional file 2: Table S1). To construct a control set of tumors that were HR-proficient, we excluded tumors with high large-scale state transition (LST), signature 3 exposures (Signature 3 is a distinct pattern of single-base substitutions (SBS) associated with inactivation of BRCA1/BRCA2 [52]), or a mono-allelic genetic alteration in *BRCA1*/*BRCA2* (Fig. 1a; the “Methods” section). Not surprisingly, *TP53* mutations were significantly enriched in bBRCA1/2 OV compared to the controls (*P* = 0.017). The only other gene significantly co-mutated after correcting for false discovery was *NF1* (*P* = 0.004) in OV (Additional file 1: Fig. S2d). In ER+ BC, *PIK3CA* mutations were nominally mutually exclusive with *BRCA1*/*BRCA2* mutations (*P* = 0.01). In TNBC, *NF1* mutations more frequently occurred in *BRCA1*/*BRCA2* tumors (*P* = 0.03). As expected, genes including 53BP1 and PAXIP1 previously identified in murine models as synthetically viable in BRCA1/BRCA2-deficient tumors were not frequently altered in human cancers (Additional file 1: Fig. S2a-c) [53, 54]. This result highlights the importance of identifying putative alterations from human tumors. An analysis using an expanded set of cancer-related genes (COSMIC Cancer Gene Census [36]), produced similar results with no other recurrently mutated gene significantly enriched in BRCA1/BRCA2 mutant cancers (Additional file 1: Fig. S2e).

We subsequently hypothesized that the co-occurring alterations necessary for oncogenesis in BRCA1/BRCA2 tumors might arise through a recurrent CNA, given that these tumors often exhibit extensive genomic changes. As anticipated, bBRCA1/2 tumors displayed a higher proportion of their genome altered in ER+ BC, and TNBC than control tumors, and showed a trend towards a higher fraction of genome altered in OV tumors (Additional file 1: Fig. S2f). Similar to our analysis of mutations, we sought to identify specific CNAs enriched in bBRCA1/2 tumors. Given that bBRCA1/2 tumors are known to produce copy number and structural alterations that typically span megabases in size [6], we focused on analyzing cytobands across the genome and compared rates of amplification and deletion between bBRCA1/2 tumors and their wild-type controls (Fig. 1a; see the “Methods” section). We identified 148 cytobands (23 distinct genomic loci) more frequently deleted in OV and 90 cytobands in ER+ BC bBRCA1/2 tumors (15 distinct genomic loci) than in their wild-type controls, after adjusting for multiple testing (Fig. 1b, c; FDR < 0.05; Additional file 4: Table S3a-b). Amplifications enriched in bBRCA1/2 tumors were less frequent than deletions, with 10 and 39 cytobands located in 1 and 9 distinct genomic loci identified in OV and ER+ BC, respectively, the majority of which were near the previously described MYC locus [55] (Fig. 1d, e; Additional file 4: Table S3c-d). No cytoband exhibited enrichment for amplification or deletion in TNBC (Additional file 1: Fig. S3a), likely due to limited sample size and the small number of bBRCA1/2 tumors in this breast cancer subtype (*n* = 18).

The enriched cytobands were not only altered in bBRCA1/2 tumors but were more frequently altered in those tumors compared with wild-type controls. Moreover, these enriched segments often co-occurred (Additional file 1: Fig. S3b-c). A comparison of OVs and ER+ BCs identified twenty-six and eight cytobands that were either deleted or amplified, respectively, in common between the two cancer types (Additional file 4: Table S3e). To account for the increased genome instability typically observed in bBRCA1/2 tumors, we performed a permutation test to evaluate whether the observed cytobands enriched in bBRCA1/2 tumors were solely a result of their increased levels of CNAs overall. This analysis maintained the number of segments deleted per case and the frequency of deletions per cohort constant and demonstrated a higher number of enriched segments than expected by chance (empirical *P* < 0.00001; Additional file 1: Fig. S4a-b; see the “Methods” section). This observation is consistent with the notion that our findings are not merely a byproduct of increased genomic instability [33, 34].

To assess the generalizability of the enriched loci we identified, we obtained whole-genome sequencing data from 69 OVs, 320 ER+ BCs, and 162 TNBCs from ICGC. Even with a significantly reduced number of tumors, among the recurrently deleted cytobands we discovered in TCGA, we found that 72 and 83 deleted cytobands were significantly different (*P* < 0.1) compared to histology-matched controls (Fig. 1f, g, Additional file 1: Fig. S5a-e). In total, these results suggest bBRCA1/2 tumors are indeed associated with the deletion of specific loci in the cancer genome. Collectively, these loci encompass over 4,500 genes that could potentially be in a synthetic viable interaction with bBRCA1/2 in human cancers. Pathway enrichment analysis further revealed that these genes were involved in diverse pathways, including transcription, cell cycle, RNA metabolism, G protein-coupled receptors (GPCR) signaling, and Rho GTPases, among others (Additional file 1: Fig. S6).

### Genes in recurrently deleted loci in BRCA1/BRCA2 tumors are more likely tumor suppressors and transcriptionally downregulated

Somatic copy number alterations are known to influence transcriptional levels of genes significantly [56, 57]. Consistent with this notion, genes located within deleted loci enriched in bBRCA1/2 cancers were typically found to have lower expression levels directly correlated with gene copy number (Additional file 1: Fig. S7). Therefore, we posited that genes responsible for mediating synthetic viability, located in these recurrently deleted loci, should consistently exhibit decreased expression.

**Fig. 2:**
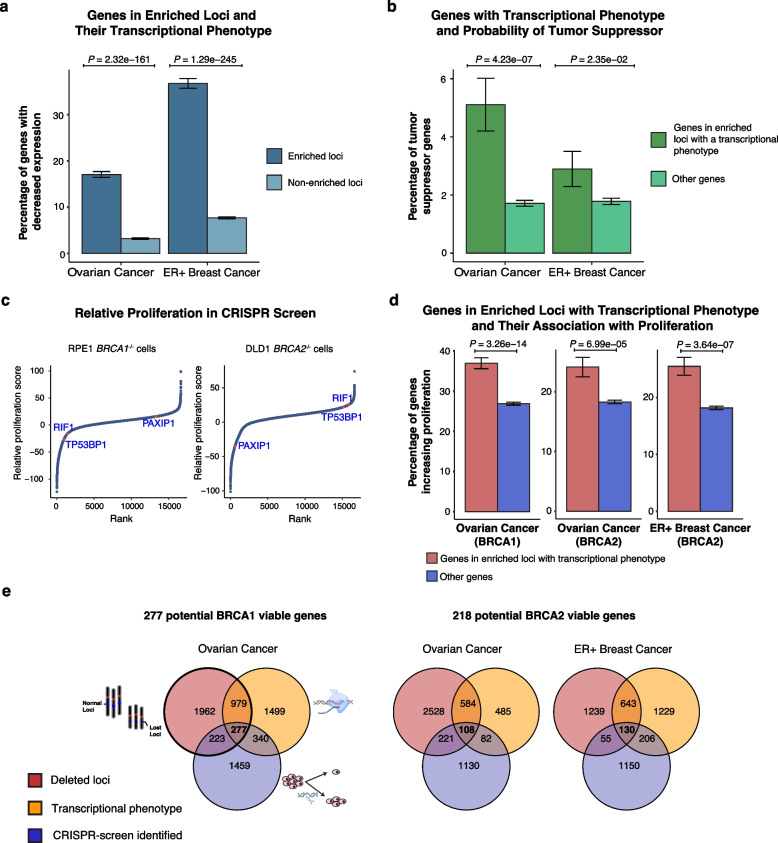
Genome-wide CRISPR screen defines transcriptionally decreased copy number deletion genes potentially increasing BRCA1 or BRCA2 cell proliferation. **a** Genes in the enriched loci with deletions and their transcriptional phenotype. The frequency of transcriptionally decreased genes was compared between genes in the enriched and non-enriched loci. *P*-value was obtained using the two-sided Fisher’s exact test. Error bars represent standard errors estimated using a proportion test. **b** Genes with transcriptional phenotype and probability of tumor suppression. The frequency of tumor suppressor genes between genes in the enriched loci with a transcriptional phenotype (decreased expression) and other genes were compared. *P*-value and error bars as in **a**. **c** Rank plots of the gene relative proliferation score in RPE1 *BRCA1*^−/−^ cells and DLD1 *BRCA2*^−/−^ cells. The relative proliferation score represents the subtracted Bayes factor (BF) score between *BRCA1*^−/−^ and WT cells or *BRCA2*^−/−^ and WT cells. For example, a known synthetic viability gene, *TP53BP1*, has a negative proliferation score in RPE1 *BRCA1*^−/−^ cells. **d** Genes in the enriched loci with transcriptional phenotype and their association with proliferation. The genes in the enriched loci with deletions and transcriptional phenotype displayed more genes increasing proliferation than other loci. Analysis in ER+ BRCA1 mutated tumors was not conducted due to a small number of cases (*n* = 5). The *P*-value was calculated by the hypergeometric test. **e** Scheme for identifying candidate synthetic viability genes affected by copy number deletions in bBRCA1/2 tumors. Recurrent copy number deletion loci or genes were first identified; then, their gene expression levels were further evaluated to identify transcriptionally decreased genes. Using a genome-wide CRISPR screen, the genes increasing proliferation were thus selected. BRCA2 viable genes identified in OV and ER+ BC were combined

We first performed standard differential gene expression analysis between bBRCA1/2 and histology-matched controls in OV and ER+ BC and identified 623 and 388 differentially expressed genes (DEGs, FDR < 0.05), respectively (Additional file 1: Fig. S8a-b). The majority of DEGs were downregulated (70–80%), and, unsurprisingly, these genes were more frequently located within loci enriched in bBRCA1/2 tumors (*P* = 7.2e − 7 for OV, *P* = 4.2e − 7 for ER+ BC, Fisher’s exact test; Additional file 1: Fig. S8c-e). As the incidence of deletion of these loci varied in bBRCA1/2 cancers (range: 13–88%, median 55%), a standard differential expression analysis across the entire bBRCA1/2 cohort might underestimate genes within deleted loci that exhibit corresponding transcriptional evidence of downregulation. To address this, we introduced a transcriptional consistency score (TCS) to evaluate how frequently the bBRCA1/2 tumors with the deletion of a gene consistently displayed decreased expression (below the cohort median; see the “Methods” section, Additional file 1: Fig. S9a). Using this approach, we identified 587 and 760 genes located within the enriched loci with deletions in OV and ER+ BC, respectively (Additional file 5: Table S4). The TCS analysis, by considering which tumors had deletions of specific genomic loci, increased the number of transcriptionally decreased genes in enriched loci by 4- to tenfold compared to standard DEG analysis. When applying TCS analysis across the genome, we found that genes in enriched loci were more likely to exhibit consistent decreased expression (Fig. 2a and Additional file 1: Fig. S9b). These analyses suggest that deletions within enriched loci have a more significant phenotypic effect than deletions in other regions of the genome. Lastly, the incorporation of TCS with our copy number analysis to identify putative synthetic viability genes revealed a significant enrichment of tumor suppressor genes, as previously defined by Davoli et al. [25] (Fig. 2b). Taken together, these results suggest that the enriched loci with recurrent copy number deletions have a consistent transcriptional phenotype.

### Human genomic analysis identifies genes that increase viability in CRISPR/Cas9 screens

We subsequently sought to identify the most promising synthetic viability candidates by integrating cancer genomic and transcriptomic analyses with results from recent genome-wide CRISPR knockout screens in *BRCA1* and *BRCA2* knockout isogenic cell lines [42]. While these experiments are commonly performed to identify synthetically lethal candidates, they can also reveal genes that increase viability [58, 59]. To this end, we reanalyzed recently published data on a BRCA1 knockout in RPE1 and a BRCA2 knockout in DLD1 [42] using the BAGEL algorithm [41]. Briefly, BAGEL employs a Bayesian approach to identify synthetically lethal and essential genes and computes a Bayes factor (BF) score to evaluate gene essentiality [41]. As anticipated, we observed *PARP1*, *APEX2*, and *FEN1* have synthetically lethal interaction with both BRCA1 and BRCA2 (Additional file 1: Fig. S10a) [42, 60–62]. To ensure the reliability of the previously published screen, we repeated a genome-wide CRISPR screen in BRCA2 knockout in DLD1, and BF scores from our CRISPR screen in DLD1 and those from the screen performed by Alvarez-Quilon et al. [42] were strongly correlated (Pearson’s *r* = 0.7, Additional file 1: Fig. S10b; Additional file 6: Table S5 for BF scores).

To identify genes whose loss is likely to increase cell proliferation in a bBRCA1/2 context (similar to 53BP1 for *BRCA1*^−/−^ cells), we calculated a relative proliferation score (RPS) for each gene by subtracting WT BF score from the BF score of *BRCA1*^−/−^ or *BRCA2*^−/−^ cells. Relative proliferation (negative scores suggesting that inhibiting genes increased proliferation in *BRCA1*^−/−^ or *BRCA2*^−/−^ cells) identified previously known genes to improve cellular viability such as 53BP1 [53] (Fig. 2c). Given that the CRISPR screens were performed separately for *BRCA1*^−/−^ and *BRCA2*^−/−^ backgrounds, we reanalyzed human cancer genomic data separating BRCA1 and BRCA2 tumors in OV and ER+ BC to identify candidate drivers for each gene individually (Additional file 1: Fig. S11a-b). Subsequently, we identified candidate synthetic viability genes by selecting those that increased proliferation in *BRCA1*^−/−^ and *BRCA2*^−/−^ CRISPR experiments separately. We observed that genes located within enriched loci with a transcriptional phenotype identified above are more likely to increase proliferation than other genes in the genome (Fig. 2d).

To ascertain the most promising synthetically viable genes within the enriched loci affected by deletions for experimental validation, we implemented a more stringent selection procedure (see the “Methods” section; Additional file 1: Fig. S11c for illustration) and then cross-referenced these candidates with our genomic analysis of human tumors (Fig. 2e). Using this approach, we reduced our list of synthetically viable candidates to 277 genes for BRCA1 knockdown cells and 218 genes for BRCA2 knockdown cells (Additional file 7: Table S6). Using a de-convolution approach with a single-cell RNA-sequencing reference to identify gene expression of individual genes originating from cancer cells resulted in similar results, with over 90% of originally identified candidates validated (Additional file 1: Fig. S12).

Analysis of genes in common between BRCA1 and BRCA2 that facilitate synthetic viability revealed 75 genes that were enriched for pathways involving RNA transcription, cell cycle, TP53 regulation, and immune pathways (Additional file 1: Fig. S13a-b). The majority of genes identified were unique to BRCA1 or BRCA2, although they often occurred in similar pathways, albeit with a notable increase in genes involved in nuclear envelope reassembly identified in BRCA1 (Additional file 1: Fig. S13b).

To further evaluate the likelihood of synthetic viability of our 277 BRCA1 candidates, we turned our attention to DepMap, which has performed genome-wide CRISPR analysis in 1100 cancer cell lines. We focused on three of these lines, which were known to have bi-allelic alterations for *BRCA1*. Of the 277 candidate genes, 128 had evidence to suggest an increase in proliferation (Additional file 8: Table S7). However, comparing known positive controls (e.g., *TP53BP1*, *TP53*) and established negative controls (*PARP1*, *POLQ*) suggests that results from DepMap may be dampened due to a lack of isogenic controls and a short-term proliferative readout (Additional file 1: Fig. S14).

### ATMIN and DYNLL1 are synthetically viable in bBRCA1 cells and recurrently lost in human cancers

**Fig. 3:**
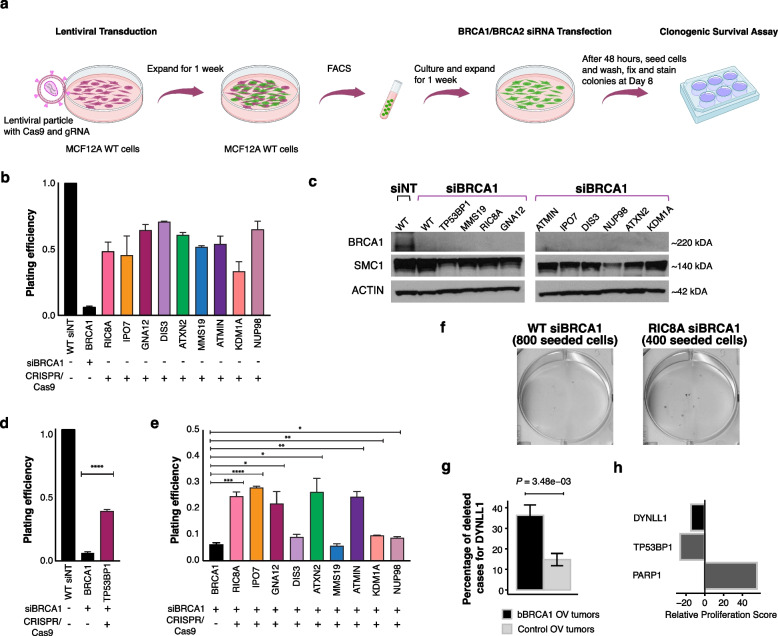
Evaluation of cell survival for BRCA1 candidate genes using clonogenic assay. **a** Schematic of experimental validation (see the “Methods” section). FACS, fluorescence-activated cell sorting; siNT, non-targeted siRNA; siBRCA1/2, siRNA-mediated BRCA1 or BRCA2 knockdown. Created with BioRender.com. **b** Plating efficiency of knockdown of candidate genes normalized to WT siNT cells (*n* = 3 biological replicates, mean values with error bars representing s.e.m). **c** Western blot of BRCA1 after siRNA knockdown. **d** Plating efficiency of siBRCA1 cells with edited TP53BP1 (*n* = 3 biological replicates). Plating efficiency was normalized same as **b**. **e** Plating efficiency of siBRCA1 cells with candidate genes edited (*n* = 3 biological replicates, *P < 0.05, **P < 0.01, ***P < 0.001, ****P < 0.0001, the two-sided Student’s *t*-test). Plating efficiency was normalized as in **b**. **f** Representative clonogenic survival plates. **g** Frequency of DYNLL1 deletion in bBRCA1 OV tumors and control tumors. *P*-values were obtained from cytoband enrichment analysis based on the two-sided Fisher’s exact test followed by multiple testing corrections as described in the “Methods” section. Error bars represent standard errors estimated using a proportion test. **h** Relative proliferation score of DYNLL1 for *BRCA1*^−/−^ vs. WT cells. TP53BP1 increases proliferation and PARP1 decreases proliferation are shown

We next sought to experimentally validate the computationally identified set of synthetically viable candidate genes within genomic loci enriched for deletions in bBRCA1/2 cancers. We selected nine genes linked to BRCA1 deficiency to test for synthetic viability using an orthogonal system evaluating cellular survival during early oncogenesis by using an immortalized non-cancerous breast epithelial cell line, MCF-12A, which is TP53 proficient. Briefly, we transfected MCF-12A cells with CRISPR/Cas9 and a guide to knock out a candidate gene. Subsequently, we transfected these cells with a siRNA targeting BRCA1 or BRCA2. These cells then underwent a clonogenic survival assay to evaluate their ability to form colonies (see the “Methods” section; Fig. 3a).

Sequencing analysis of nine BRCA1 candidates and 53BP1 (used as a positive control) after CRISPR/Cas9 transfection revealed a high frequency of gene inactivation (Additional file 1: Fig. S15). The depletion of these candidate genes did not increase plating efficiency in an HR-proficient setting (Fig. 3b). Subsequently, cells were transfected with a siRNA against BRCA1, and successful knockdown was confirmed by western blot analysis (Fig. 3c). The analysis of the previously described synthetically viable relationship with 53BP1 and BRCA1 in the same experimental system demonstrated a marked enhancement in colony formation (Fig. 3d). Of the nine candidate genes (see the “Methods” section for selection criteria), seven genes exhibited significantly increased viability, namely *GNA12*, *RIC8A*, *ATMIN*, *IPO7, ATXN2*, *KDM1A*, and *NUP98* (Fig. 3e, f). For these validated genes, most of the deletions in BRCA-mutant tumors were heterozygous (Additional file 9: Table S8).

Of particular significance, ATMIN (ATM-INteracting protein, also known as ASCIZ (ATM/ATR substrate Chk2-interacting Zn^2+^-finger protein)) was frequently deleted in bBRCA1 ovarian cancers, with a prevalence of 68%. Previous studies have demonstrated that ATMIN plays a critical role in initiating ATM-mediated signaling and recruitment of 53BP1 to DNA damage sites [48]. Moreover, ATMIN can modulate end-resection through its regulation of DYNLL1 [63]. Loss of ATMIN leads to depletion of DYNLL1 and restores HR in *BRCA1*-mutant cells [63]. Furthermore, the loss of these two genes has also been implicated in resistance to cisplatin and PARP inhibitors. Interestingly, DYNLL1 was also situated within a genomic locus enriched for deletion (12q24.31) in bBRCA1 OV cancers (deleted in 36% vs. 15% in control tumors; *P* < 3.48e − 3, Fig. 3g). Not surprisingly, we also observed DYNLL1 increased proliferation using our relative proliferation score metric (Fig. 3h). Collectively, these results support the notion that integrative analysis of human genomics with CRISRP/Cas9 screens can identify phenotypically relevant genes from large copy number alterations in cancer.

### RIC8A-inactivation is synthetically viable with both BRCA1 and BRCA2 mutations and may affect mitosis

**Fig. 4:**
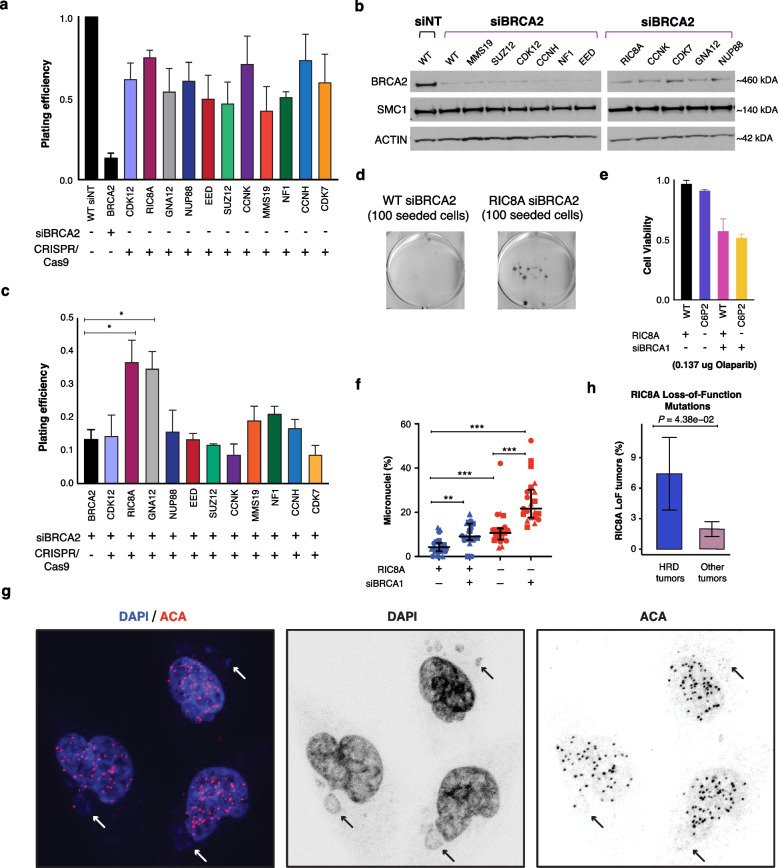
RIC8A is synthetically viable with BRCA2 and recurrently mutated in HRD tumors. **a** Plating efficiency of knockdown of candidate genes normalized to WT cells (*n* = 3 biological replicates, mean values with error bars representing s.e.m). **b** Western blot of BRCA2 after siRNA knockdown. **c** Plating efficiency of siBRCA2 cells with candidate genes edited (*n* = 3 biological replicates, mean values with error bars representing s.e.m; *P < 0.05, Student’s *t*-test). Plating efficiency was normalized same as **b**. **d** Representative clonogenic survival plates. **e** Cell viability in RIC8A and BRCA1 knockdown MCF12A cells treated with olaparib PARP inhibitor. **f**, **g** Quantification of micronuclei in cells of indicated genotype and siRNA treatment and representative image of micronuclei in RIC8A KO with siBRCA1 treatment, respectively. ** *P*-value < 0.01, *** *P*-value < 0.001, two-sided Mann–Whitney *U* test. **h** Evaluation of *RIC8A* loss-of-function mutations in HRD tumors and other tumors. *P*-value was obtained using the two-sided Fisher’s exact test

We then conducted a similar analysis of 11 candidate genes associated with synthetic viability for BRCA2. As observed with the BRCA1 candidates, none of these genes displayed an increase in proliferation in an HR-proficient setting (Fig. 4a). Subsequently, cells were transfected with siRNA against BRCA2 to investigate synthetic viability, and successful knockdown was confirmed via western blot (Fig. 4b). Of the 11 candidates (see the “Methods” section for selection criteria), silencing of *RIC8A* and *GNA12* resulted in an increase in proliferation (Fig. 4c, d). Both genes were also identified as synthetic viable partners of *BRCA1* (Fig. 3e; Fig. 4c)*.*

To elucidate the mechanism by which RIC8A leads to synthetic viability, we first evaluated if it may partially rescue an HR-deficient phenotype, similar to *TP53BP1.* We used CRISPR/Cas9 to create *RIC8A*^−/−^ MCF12A single-cell clones. Evaluation of cell viability after PARP inhibition, which is synthetically lethal in the context of HRD, did not demonstrate improved viability in *RIC8A*^−/−^/siBRCA1 MCF12A cells compared to siBRCA1 cells, suggesting partial rescue of HRD is unlikely to be the mechanism of synthetic viability. (Fig. 4e; Additional file 1: Fig. S16).

Interestingly, RIC8A has been shown to play a crucial role in regulating mitotic spindle pole movement and acts as a molecular chaperone, typically as a guanine nucleotide exchange factor, facilitating the folding of G protein α subunits [64, 65]. Prior work has identified that RIC8A interacts with and stabilizes Gα12 (the protein product of *GNA12*) [66]. The expression of RIC8A has been shown to increase the protein level of Gα12 [66]. Hence, we used our *RIC8A*^−/−^ MCF12A cells to evaluate whether they influenced mitosis in BRCA1-null cells. By flow cytometry, we observed a slight increase in cells that were in mitosis in the dual knockouts compared to BRCA1-null cells alone (Additional file 1: Fig. S17). Surprisingly, we observed a significant increase in micronuclei, a downstream consequence of mitotic errors, in *RIC8A*^−/−^/siBRCA1 MCF12A cells, compared to a single knockout (Fig. 4f, g). Together, these data suggest that synthetic viability between BRCA1 and RIC8A occurs from an interaction in mitosis, although further work will be necessary to elucidate the entire mechanism.

### RIC8A is recurrently mutated in metastatic HR-deficient breast **cancer**

Mutations in *RIC8A* have been recently identified to be enriched in metastatic breast cancer [51]. To explore whether these mutations are related to HR deficiency, we reanalyzed data from Bertucci et al. [51] and found that a majority of these mutations in ER+ BC were loss-of-function mutations (48% of 23 mutations). This is consistent with the potential tumor suppressor roles of RICA8 and the potential impact of copy number losses affecting this gene in bBRCA1/2 cancers [24, 25]. Consistent with our findings, we observed that *RIC8A* loss-of-function mutations were four times more likely to occur in HR-deficient ER+ BC (7.4%) than in wild-type controls (2.0%) in this cohort (see the “Methods” section, Fig. 4h, *P* = 0.04, Fisher’s exact test). Taken together, these findings support that a RIC8A-GNA12-mediated process likely plays a role in oncogenesis in HR-deficient malignancies.

## Discussion

BRCA1/BRCA2, established tumor suppressors, have been observed paradoxically to inhibit cellular proliferation, which has been difficult to reconcile with their known cancer predisposition phenotype [7, 9]. BRCA1/BRCA2-associated cancers are characterized by genomic instability, leading to large-scale copy number alterations. The genes specifically selected by these alterations remain to be elucidated. Here, we coupled high-throughput genomic analysis of human tumors with genome-wide CRISPR screening in model systems to identify genes from large-scale genomic alterations that are synthetically viable with loss of BRCA1/BRCA2 function. We validated several genes that induce synthetic viability with BRCA1 and BRCA2 deficiencies, including a previously described relationship with ATMIN and its role in end-resection [63]. In total, 40% of genes selected for validation were experimentally validated to induce synthetic viability in BRCA1 and BRCA2 (Fig. 3e; Fig. 4c).

We observed a considerable portion of the genome that exhibited a higher tendency for deletion in bBRCA1/2 cancers compared to histology-matched control tumors in OV and ER+ BC. Genes within these regions were not only likely to function as tumor suppressors [25] but also displayed a consistent transcriptional phenotype, more so than genes from other genomic loci. Prior work to identify relevant genes in copy number-altered loci primarily relied on identifying minimal common segments or evaluating breakpoints [67, 68]. Importantly, these papers illustrated that large-scale copy number changes are not mere passenger events but on a pan-cancer level selected for, and if amplified, they are enriched for oncogenes, whereas if deleted, they are enriched for tumor suppressor-like genes. Shih et al. [67] found 51 large-scale copy number deletions that were selected across cancer, supporting our findings that large-scale copy number deletions can be selected in cancer. These pan-cancer methods, however, often leave numerous genes in certain loci with unclear contributions to viability, necessitating further evaluation. By integrating genome-wide CRISPR/Cas9 screens [42] with human genetic loci, we identified an enrichment of genes that enhance viability in bBRCA1/2 tumors.

We uncovered an unexpected synthetic viable relationship between BRCA1/BRCA2 and genes involved in G-protein receptor signaling, namely *RIC8A* and *GNA12*. Interestingly, RIC8A is known to stabilize Gα12 [66] and regulates abundance of Gα13, which is another one of Gα12 family member, in mice [64], mouse embryonic fibroblasts [69], and in HEK-293 cells [70]. The expression of RIC8A can increase the protein level of Gα12 probably via direct interaction [66]. Both genes have also been previously identified to be frequently lost in human cancers [71, 72]. Here, we found these genes to be significantly more frequently deleted in bBRCA1/2-related tumors. We have also discovered that previously reported mutations in *RIC8A*, which are enriched in metastatic breast cancer, specifically, are enriched in the HR-deficient cancers [51].

While our study has its limitations, we have identified that the inactivation of RIC8A is synthetically viable with bBRCA1/2 malignancies. Further work will be necessary to elucidate the mechanistic basis of this phenomenon. RIC8A is known to regulate mitotic spindle pole function, among other functions [64, 65, 73]. Likewise, understanding the mechanisms of the other synthetic viable genes will require additional research. Unveiling these mechanisms could lead to the development of novel therapeutic strategies for bBRCA1/2 cancers [74]. Additionally, due to a limited number of bBRCA1/2 cases in the TCGA database, we might not have identified all loci enriched for CNAs in bBRCA1/2 cancers. This limitation is particularly evident in the TNBC analysis but is expected to improve as more of these tumors are sequenced. Furthermore, genes on deletions that occur at low frequency may have not sufficient power to be identified by our transcriptional analysis. Last, despite our demonstration that the inactivation of individual genes can contribute to synthetic viability with bBRCA1/2 tumors, it is also possible that a compound heterozygote phenotype may achieve the same impact on viability, as suggested by our genetic data.

## Conclusions

We have applied an integrative approach to identify genes within large-scale CNAs that drive oncogenesis through their interaction with tumor mutational genotypes. Specifically, we identified genes involved in synthetic viability interactions with BRCA1/BRCA2 in human malignancies. This approach could assist in identifying other genes that drive oncogenesis through CNAs in conjunction with other mutational genotypes, providing a comprehensive analytic paradigm to investigate novel synthetic viable interactions in the development of human cancers.

### Supplementary Information

Additional file 1: Fig S1. Evaluation of BRCA1 and BRCA2 essentiality in the published CRISPR/Cas9 screen data and gene-trap integration data. Fig S2. Genetic Alterations in BRCA1/BRCA2 mutated tumors. Fig S3. Copy number alterations in bBRCA1/2 tumors. Fig S4. Permutation analysis of copy number alterations. Fig S5. Recurrent copy number alterations in BRCA1/BRCA2 tumors in ICGC. Fig S6. Top Reactome pathways for genes located in the enriched loci with deletions. Fig S7. The gene expression level was consistent with copy number alterations in the enriched cytobands with deletions. Fig S8. Differentially expressed genes identified between bBRCA1/2 and control tumors. Fig S9. A comparison of transcriptional consistency score (TCS) in copy number deletion tumors between genes in the enriched loci and non-enriched loci. Fig S10. Bayes factor scores of genome-wide CRISPR/Cas9 screen. Fig S11. Characterization of transcriptionally decreased genes in enriched loci with deletions in separate bBRCA1 and bBRCA2 tumors. Fig S12. BRCA1- and BRCA2-related candidates promoting proliferation identified according to cancer-specific expression values imputed by BayesPrism. Fig S13. Integrative analysis of BRCA1 and BRCA2. Fig S14. Evaluation of DepMap influence of knockout of various genes in BRCA1 deficient cell lines. Fig S15. TIDE analysis of CRISPR/Cas9 editing. Fig S16. Western blot of RIC8A and BRCA1 in olaparib-treated MCF12A cells. C6P2 were RIC8A-/- MCF12A cells. Fig S17. Fraction of mitotic cells in RIC8A wild-type and knock-out cells treated with BRCA1 siRNA.

Additional file 2: Table S1: TCGA bBRCA1/2 tumor IDs and bi-allelic status of OV, ER+ BC, and TNBC.

Additional file 3: Table S2. Guide-RNA target sequences for the validated synthetic viable genes.

Additional file 4: Table S3: List of identified recurrently copy number altered loci. (a) Enriched cytobands with deletions in OV. (b) Enriched cytobands with deletions in ER+ BC. (c) Enriched cytobands with amplifications in OV. (d) Enriched cytobands with amplifications in ER+ BC. (e) Common cytobands in OV and ER+ BC.

Additional file 5: Table S4. Transcriptional decreased genes in the enriched loci with deletions. Genes identified in (a) the combined bBRCA1/2 tumors in OV, (b) the combined bBRCA1/2 tumors in ER+ BC, (c) the bBRCA1 tumors in OV, (d) the bBRCA2 tumors in OV, and (e) the bBRCA2 tumors in ER+ BC.

Additional file 6: Table S5. Bayes factor scores of CRISPR/Cas9 screens.

Additional file 7: Table S6. Genes increasing cell proliferation in *BRCA1*−-/−- or *BRCA2*−-/−- cells compared with wild-type cells. (a) Genes identified from *BRCA1*−-/−- RPE1 cells. (b) Genes identified from *BRCA2*−-/−- DLD1 cells.

Additional file 8: Table S7. DepMap gene effect values for candidate synthetic viable genes with BRCA1.

Additional file 9: Table S8. Frequency of heterozygous and homozygous loss for clonogenic assay-validated genes in ER+ breast cancer and ovarian cancer that is HRD vs. HR proficient.

## Data Availability

Mutational data were obtained from the published TCGA MC3 Public MAF dataset [75]. Gene expression data were available from the TCGA GDC portal [76]. Processed CRISPR screen data are available in Additional file 6: Table S5. Raw sequencing data has been deposited to NCBI Sequence Read Archive (SRA) under the accession number: PRJNA1096436 [77]. The code used to analyze data in this paper are available at the GitHub repository [78].

## Abbreviations

bBRCA1/2: Bi-allelic inactivation of *BRCA1* or *BRCA2*
ATMIN: ATM-INteracting protein
HRD: Homologous recombination deficiency
LoF: Loss-of-function
OV: Ovarian cancer
BC: Breast cancer
TCGA: The Cancer Genome Atlas
PCAWG: Pan-Cancer Analysis of Whole Genomes working group
ICGC: International Cancer Genome Consortium
CNA: Copy number alteration
TCS: Transcriptional consistency score
RPS: Relative proliferation score
BF: Bayes factor
