# Supplementary material for: Large-scale copy number alterations are enriched for synthetic viability in BRCA1/BRCA2 tumors
Source: Genome Med. 2024 Aug 28;16:108. doi: 10.1186/s13073-024-01371-y (PMC11351199; doi:10.1186/s13073-024-01371-y)
Supplement: Supplementary file 1 — Additional file 1: Fig S1. Evaluation of BRCA1 and BRCA2 essentiality in the published CRISPR/Cas9 screen data and gene-trap integration data. Fig S2. Genetic Alterations in BRCA1/BRCA2 mutated tumors. Fig S3. Copy number alterations in bBRCA1/2 tumors. Fig S4. Permutation analysis of copy number alterations. Fig S5. Recurrent copy number alterations in BRCA1/BRCA2 tumors in ICGC. Fig S6. Top Reactome pathways for genes located in the enriched loci with deletions. Fig S7. The gene expression level was consistent with copy number alterations in the enriched cytobands with deletions. Fig S8. Differentially expressed genes identified between bBRCA1/2 and control tumors. Fig S9. A comparison of transcriptional consistency score (TCS) in copy number deletion tumors between genes in the enriched loci and non-enriched loci. Fig S10. Bayes factor scores of genome-wide CRISPR/Cas9 screen. Fig S11. Characterization of transcriptionally decreased genes in enriched loci with deletions in separate bBRCA1 and bBRCA2 tumors. Fig S12. BRCA1- and BRCA2-related candidates promoting proliferation identified according to cancer-specific expression values imputed by BayesPrism. Fig S13. Integrative analysis of BRCA1 and BRCA2. Fig S14. Evaluation of DepMap influence of knockout of various genes in BRCA1 deficient cell lines. Fig S15. TIDE analysis of CRISPR/Cas9 editing. Fig S16. Western blot of RIC8A and BRCA1 in olaparib-treated MCF12A cells. C6P2 were RIC8A-/- MCF12A cells. Fig S17. Fraction of mitotic cells in RIC8A wild-type and knock-out cells treated with BRCA1 siRNA. [file 13073_2024_1371_MOESM1_ESM.docx]

**
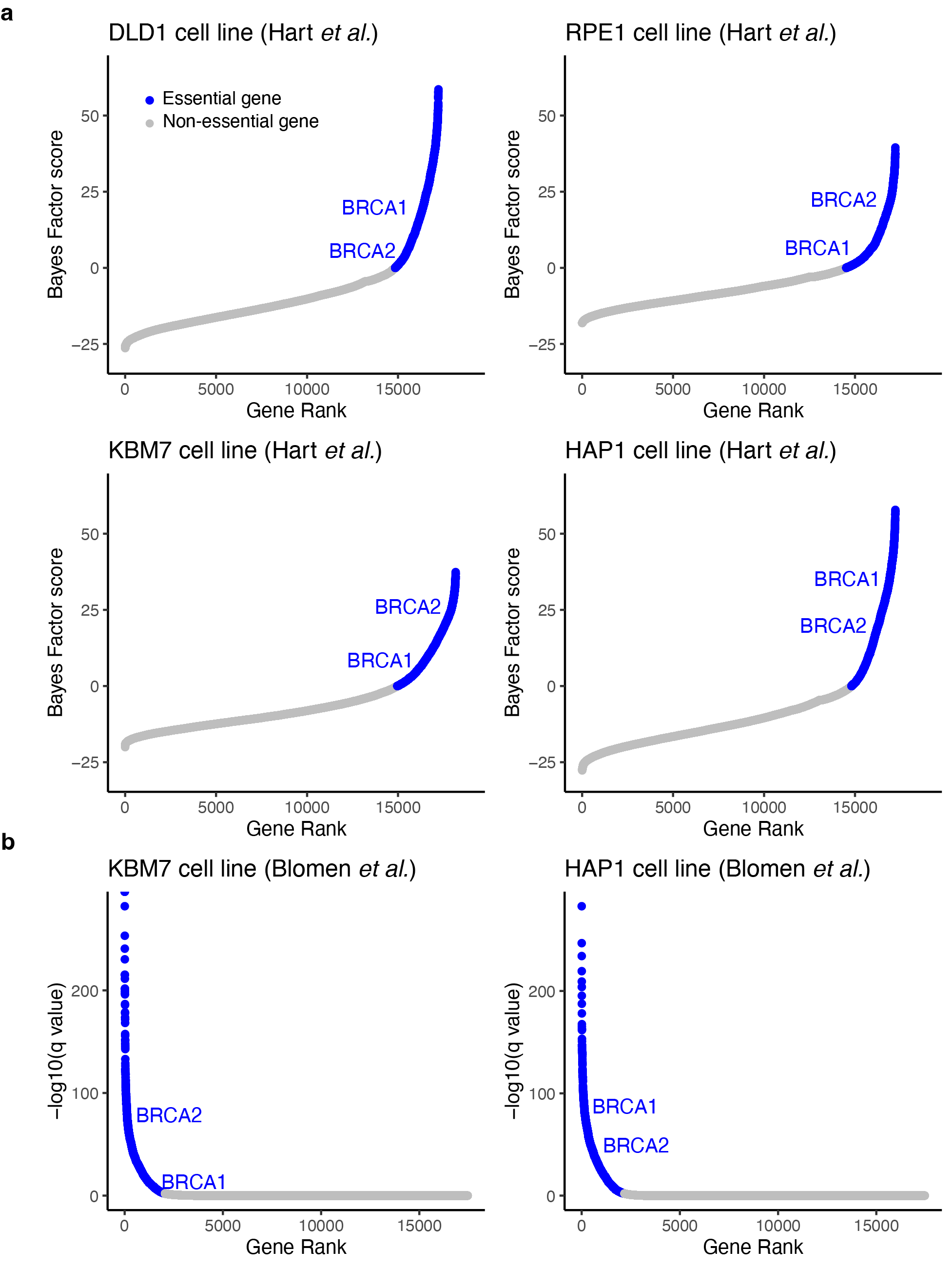
**

**Fig S1. Evaluation of BRCA1 and BRCA2 essentiality in the published CRISPR/Cas9 screen data and gene-trap integration data.** **(a)** Ranked Bayes Factor scores in CRISPR/Cas9 screen data^10^. Genes with Bayes Factor scores above zero were highlighted in blue color. **(b)** Ranked q values in gene-trap integration data^11^. Gene-trap integration was used to silence the target genes. Genes are ordered by the rank of q value, representing the enrichment of intronic integrations in the antisense direction. There are 2054 essential genes in the KBM7 cell line, and 2181 essential genes in the HAP1 cell line.

**
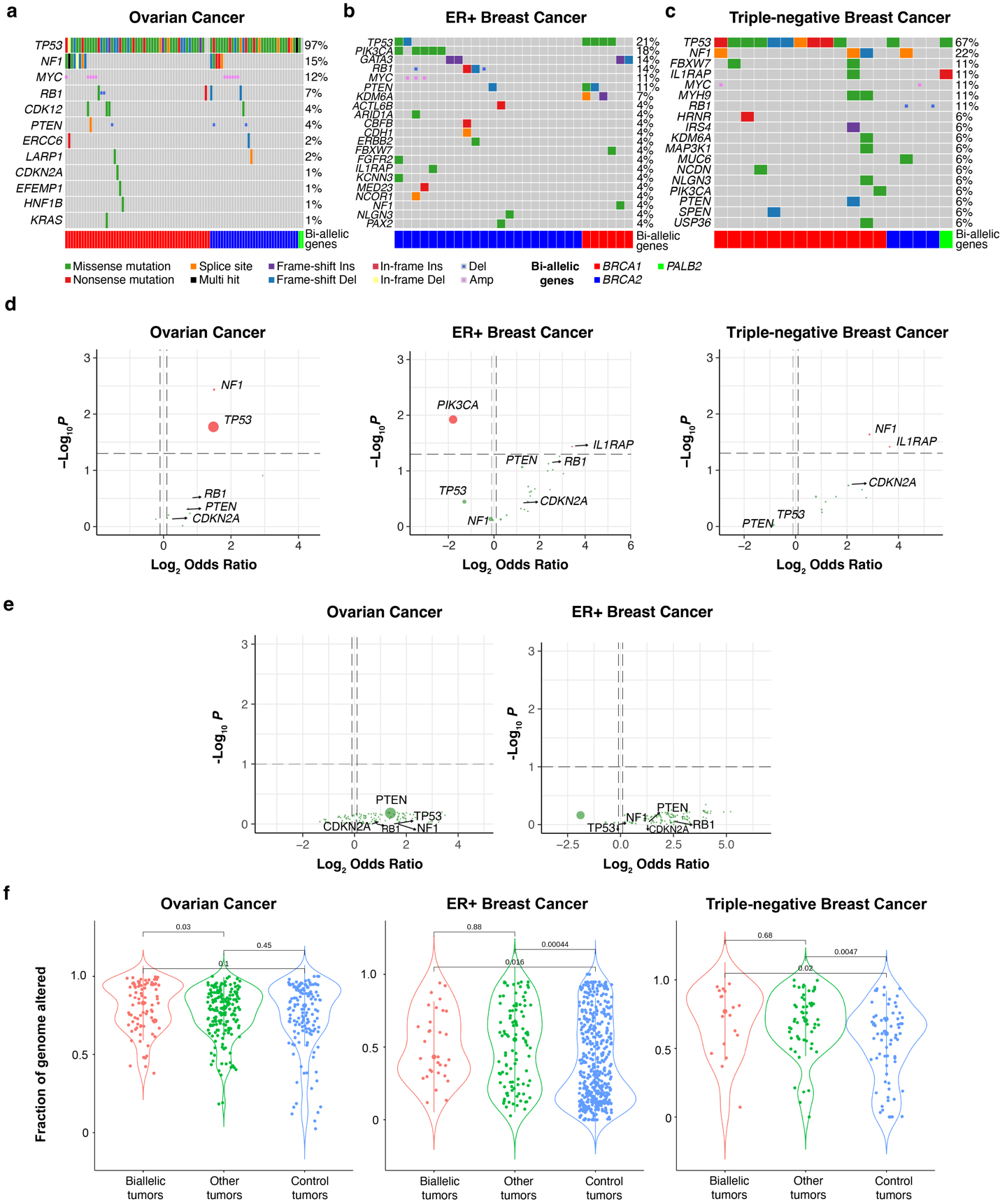
**

**Fig S2. Genetic Alterations in BRCA1/BRCA2 mutated tumors. (a)** Oncoprint of OV from TCGA with pathogenic mutations and bi-allelic loss of either BRCA1 or BRCA2 (bBRCA1/2) (n = 89). **(b)** Oncoprint of ER+ BC from TCGA with bBRCA1/2 (n = 22). **(c)** Oncoprint of TNBC from TCGA with bBRCA1/2 (n = 18). **(d)** Volcano plot of mutation enrichment in individual genes and bBRCA1/2 tumors compared to WT. *P*-values and odds ratios were calculated by the two-sided Fisher’s exact test. **(e)** Analysis of genes from the COSMIC Cancer Gene Census (572 genes) does not identify any recurrently altered gene enriched in bBRCA1/2 tumors in OV or ER+ BC (two-sided Fisher’s test with FDR adjustment). **(f)** Analysis of the fraction of genome altered (FGA) between bBRCA1/2 tumors and controls identifies that those bBRCA1/2 tumors have higher levels of copy number instability (*P*-value: two-sided Mann-Whitney U test; the dot and line in the middle of violin plot represent median and IQR values).


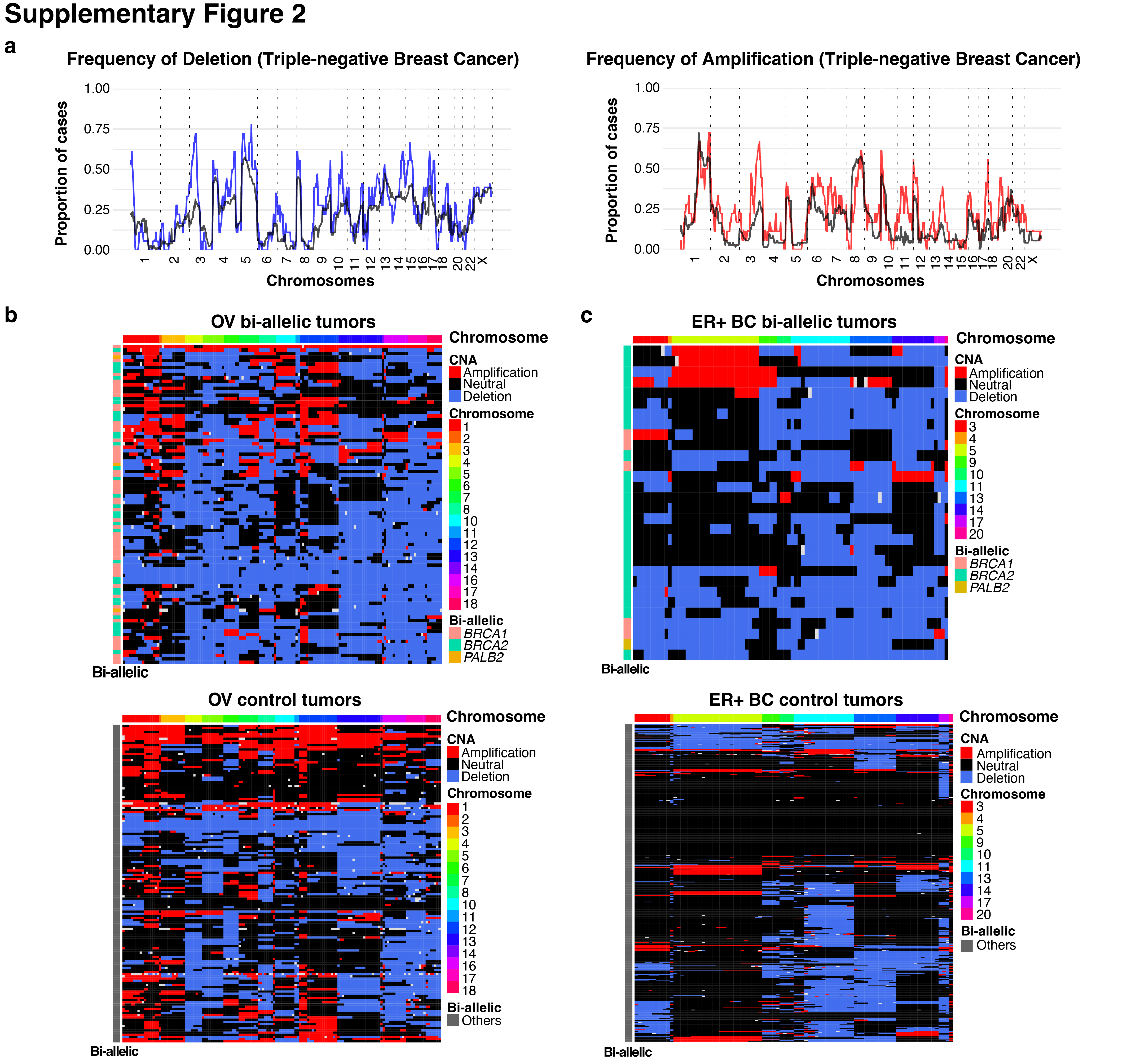


**Fig S3. Copy number alterations in bBRCA1/2 tumors. (a)** Genome-wide analysis of segments enriched for copy number deletion in bBRCA1/2 tumors compared to control tumors in TNBC identifies no cytobands enriched for amplification or deletion. **(b)** Copy number profiles are plotted for the identified 148 enriched cytobands with deletions in bi-allelic BRCA1/BRCA2 samples (top) and control samples (bottom) for TCGA OV. **(c)** Same copy number alteration profiles plotted for ER+ BC as in **(b)**. There are 90 enriched cytobands with deletions plotted for the bi-allelic tumors (top) or control tumors (bottom).

**
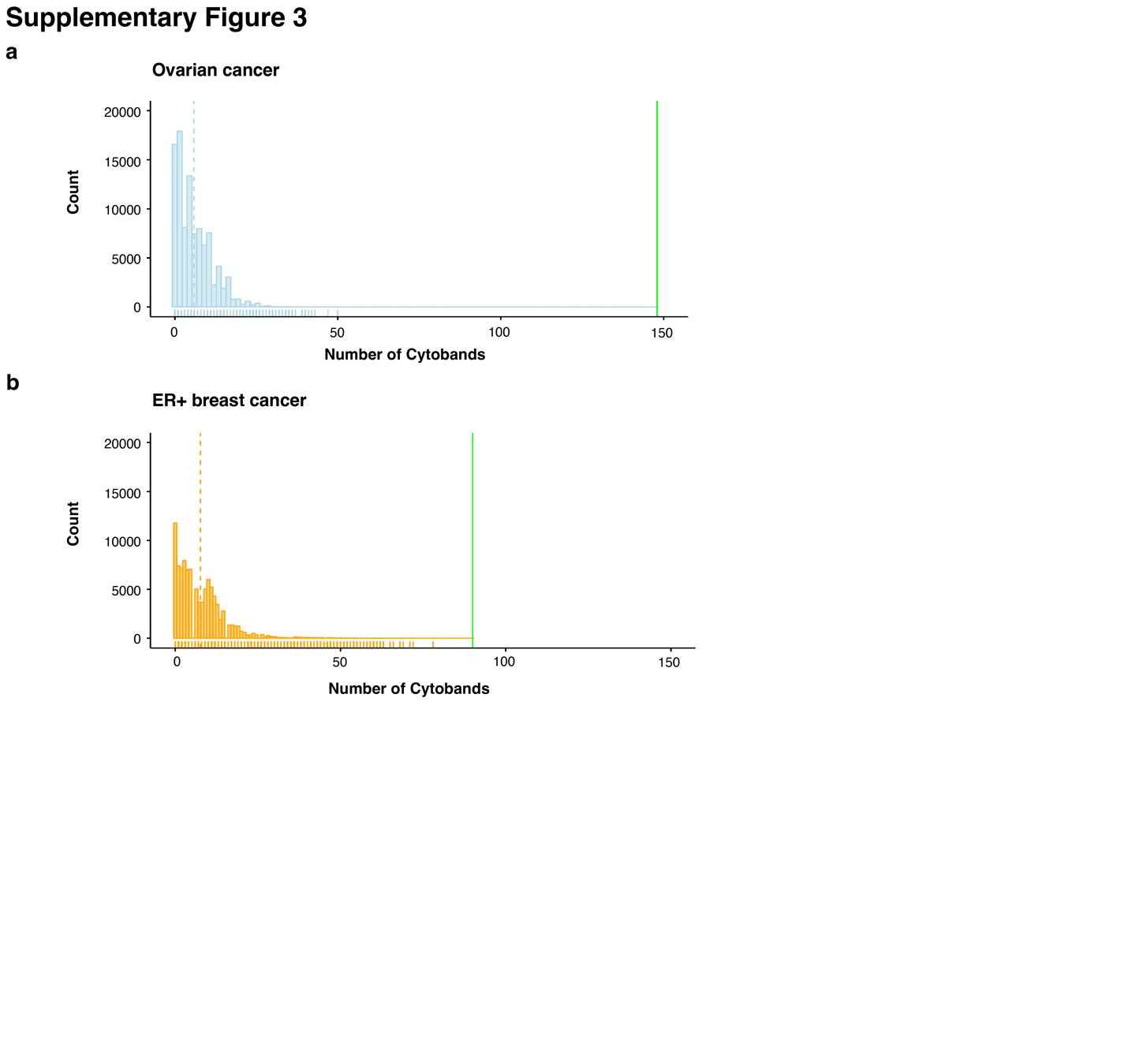
**

**Fig S4. Permutation analysis of copy number alterations. (a)** Distribution of the number of significant copy number deletion cytobands identified in permuted OV (light blue color; 100,000 permutations). The green line shows the observed number of significant copy number deletion cytobands identified (empiric *P* < 0.0001). **(b)** Similar plot as in **(a)** for ER+ BC, with the distribution of simulated copy number deletion cytoband numbers in orange (empiric *P* < 0.00001).

**
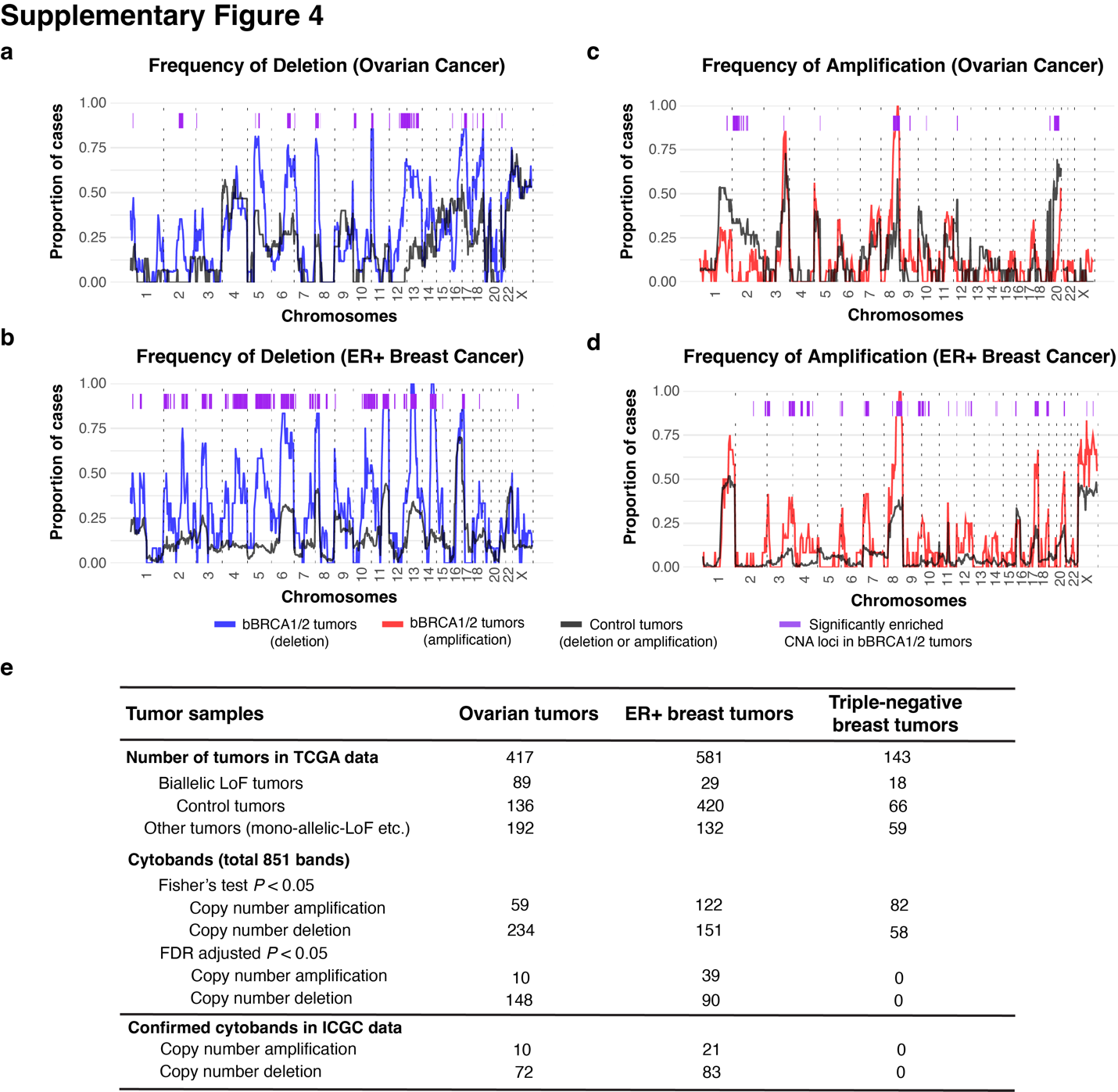
**

**Fig S5. Recurrent copy number alterations in BRCA1/BRCA2 tumors in ICGC. (a-b)** Genome-wide analysis of copy number deletion in bBRCA1/2 tumors compared to control tumors in OV **(a)** and ER+ BC **(b)**. Significant loci defined by Fisher’s exact test are highlighted in purple color. **(c-d)** Same as **(a)** and **(b)**, but for copy number amplification. **(e)** Summary table for the number of tumor samples and the number of discovered/verified recurrent copy number deletions or amplification cytobands.

**
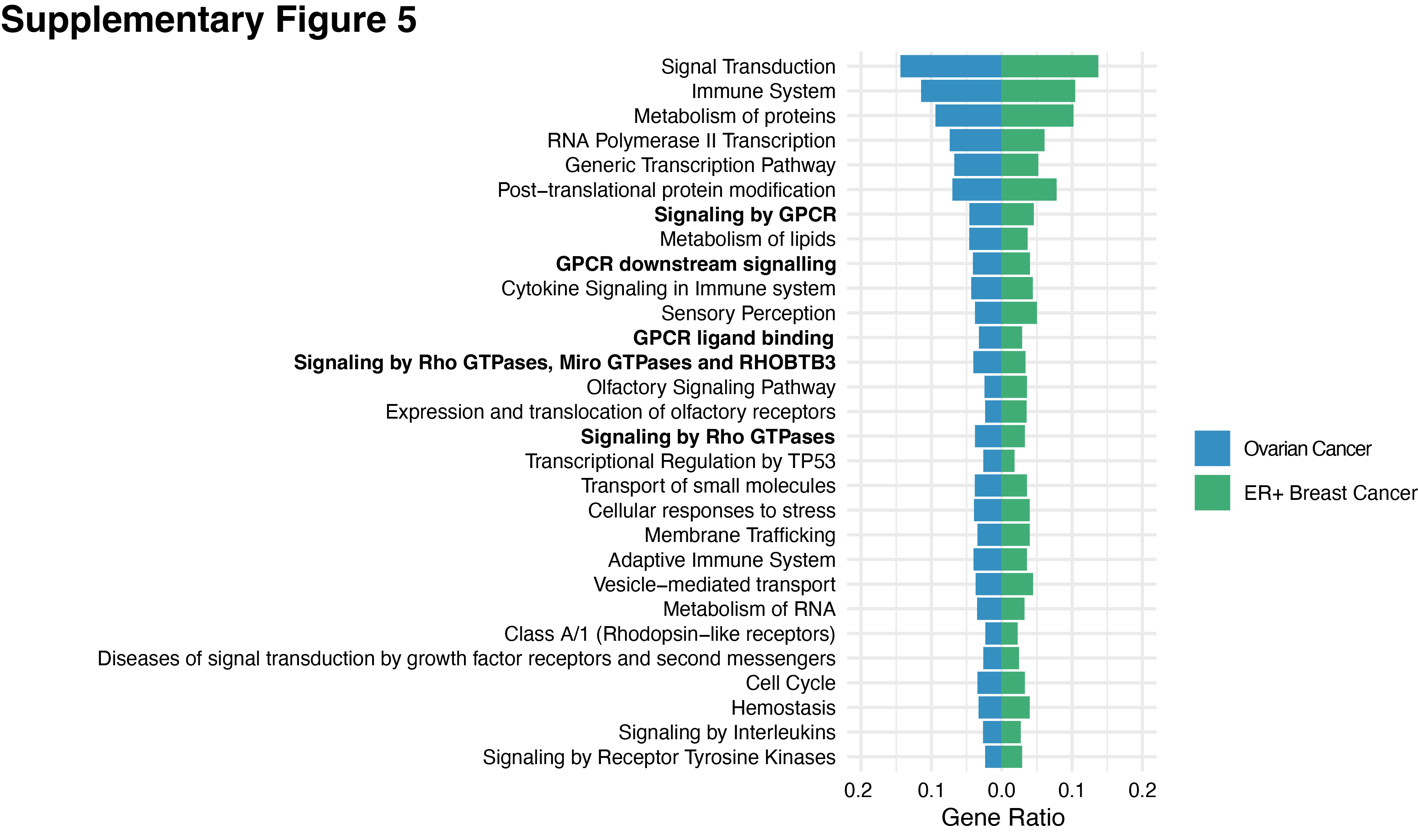
**

**Fig S6. Top Reactome pathways for genes located in the enriched loci with deletions.**

**
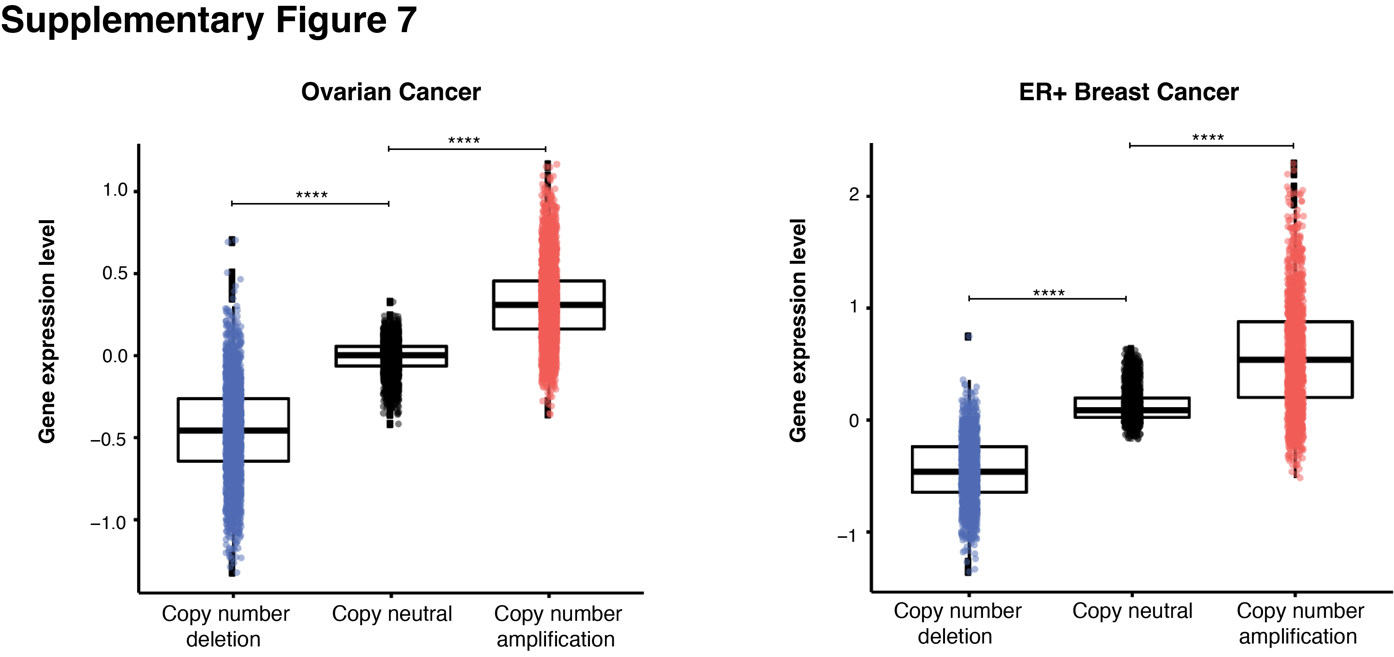
**

**Fig S7. The gene expression level was consistent with copy number alterations in the enriched cytobands with deletions.** The three horizontal lines in the boxplot proceed from top to bottom: first quartile, median, and third quartile. Each dot represents one individual gene's averaged gene expression level aggregated for patients, who either have copy number deletion, no copy number change (neutral), or copy number amplification. Only genes in the enriched loci with deletions are shown. FPKM values were transformed to Z-scores on the y-axis. **** indicates *P*-value < 0.0001 based on two-sided Mann-Whitney U test.

**
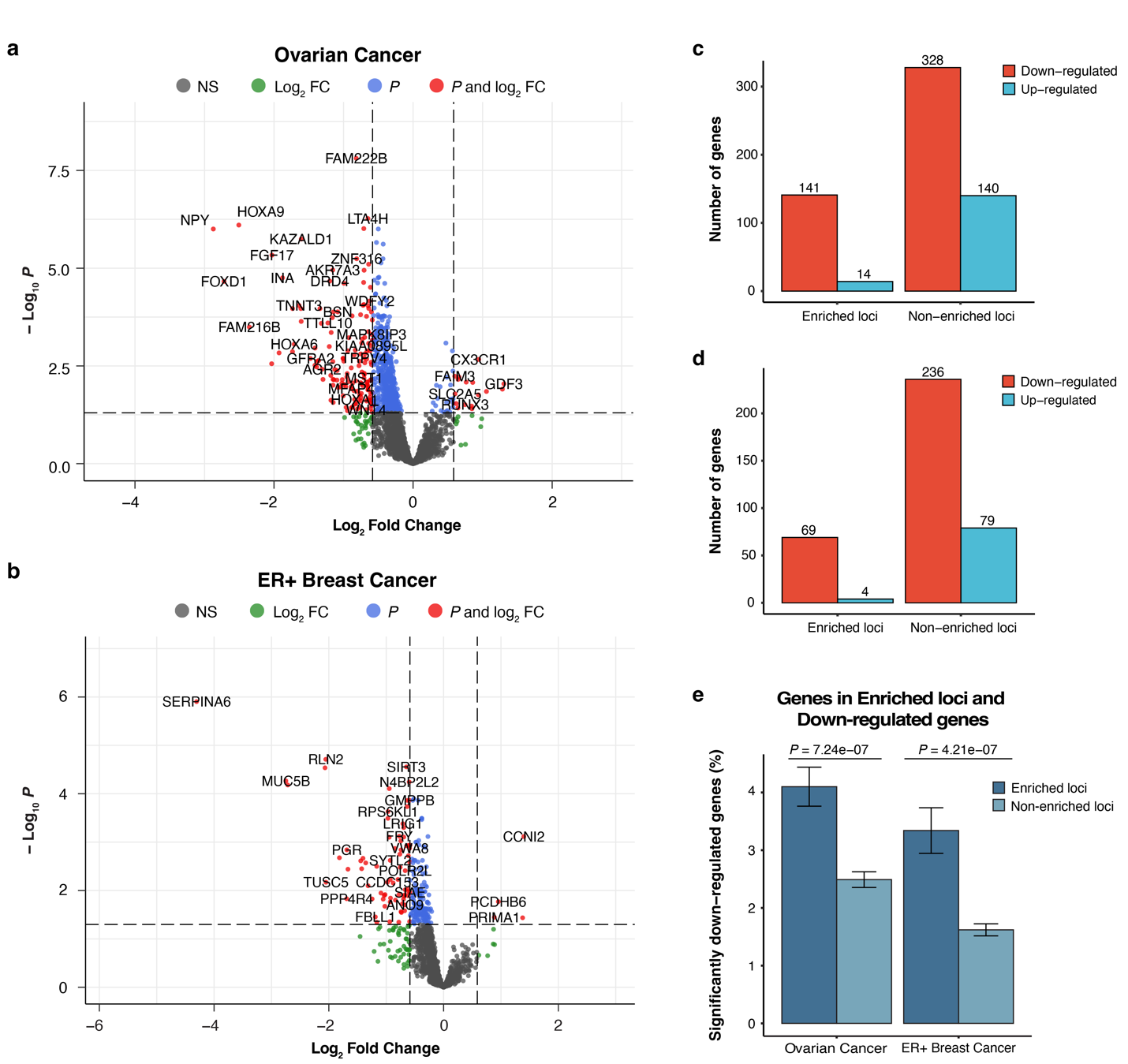
**

**Fig S8. Differentially expressed genes identified between bBRCA1/2 and control tumors.** Fold change for the genes in the enriched loci with deletions in OV **(a)** and ER+ BC tumors **(b)**. In the volcano plot, the right and left dashed lines on the x-axis represent 1.5 times higher (up-regulated) and 1.5 times lower (down-regulated) under log2 space in the comparisons of bBRCA1/2 versus control tumors, respectively. Adjusted *P* < 0.05 is considered as a significant difference between bBRCA1/2 and control tumors and marked by the dashed line on the y-axis. **(c)** and **(d)** Number of differentially expressed genes located in the enriched loci with deletions and non-enriched loci. **(e)** Enrichment of differentially expressed genes in the enriched loci with deletions. The frequency of significantly down-regulated genes was compared between the enriched and non-enriched loci. *P*-value was obtained using the two-sided Fisher’s exact test. Error bars represent standard errors estimated using a proportion test.

**
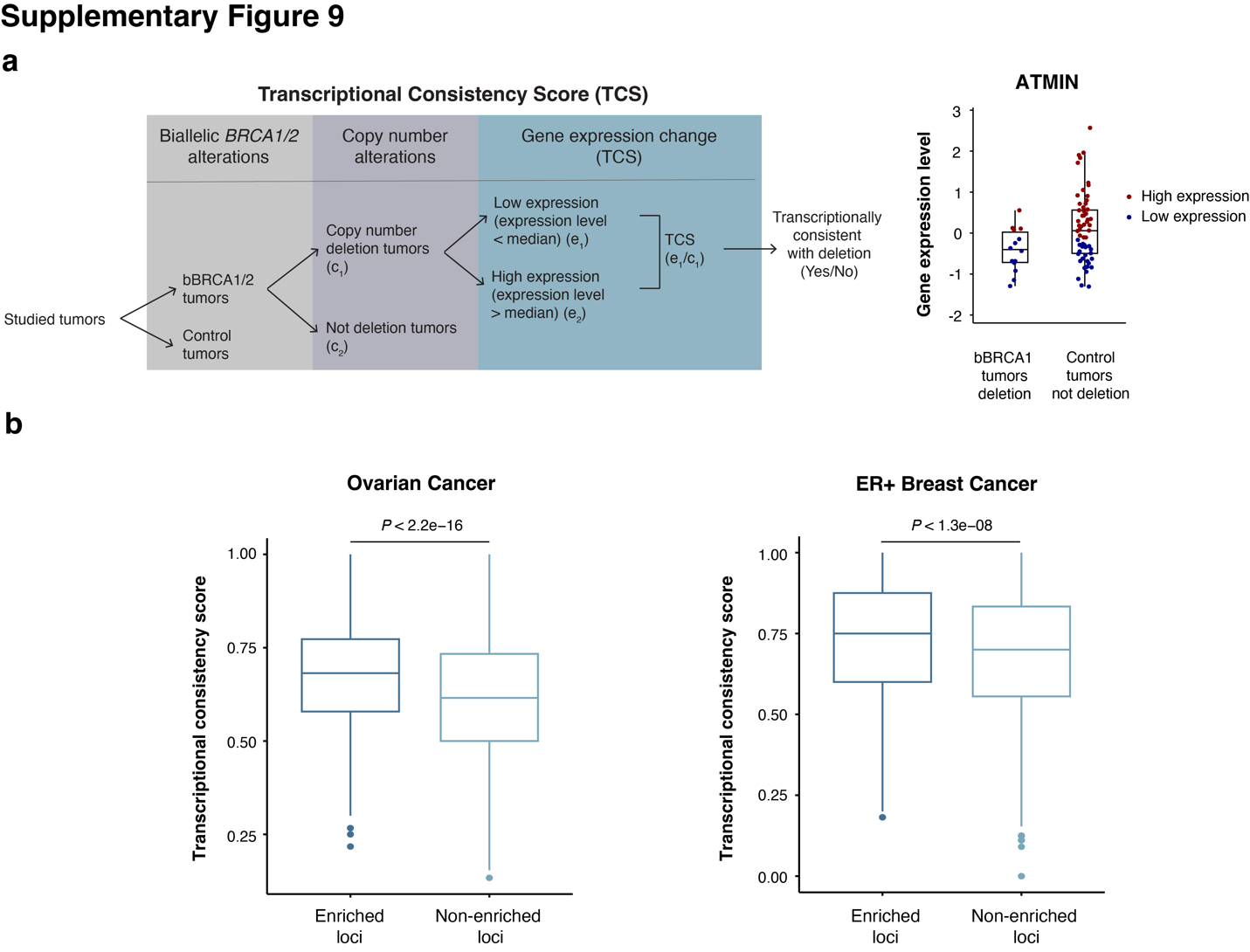
**

**Fig S9.** **A comparison of transcriptional consistency score (TCS) in copy number deletion tumors between genes in the enriched loci and non-enriched loci.** **(a)** A scheme for identifying transcriptionally consistent genes with copy number deletion. The transcriptional consistency score (TCS) for each gene was defined as the proportion of lowly expressed tumors in a group of tumors (for example, bBRCA1/2 tumors) (see **Methods** for details). The boxplot shows an example of gene expression level (FPKM value scaled to Z-score across all tumors per cancer type) for the tumors with copy number deletion in bBRCA1 tumors and not deletion in control tumors for ATMIN in OV. **(b)** The three horizontal lines in the boxplot proceeding from top to bottom: first quartile, median, third quartile. Significantly higher TCSs for genes in enriched loci compared to other genes (i.e. more consistent decreased expression, Mann-Whitney U test, *P* < 2.2e-16 for OV, *P* = 1.3e-8 for ER+ BC).


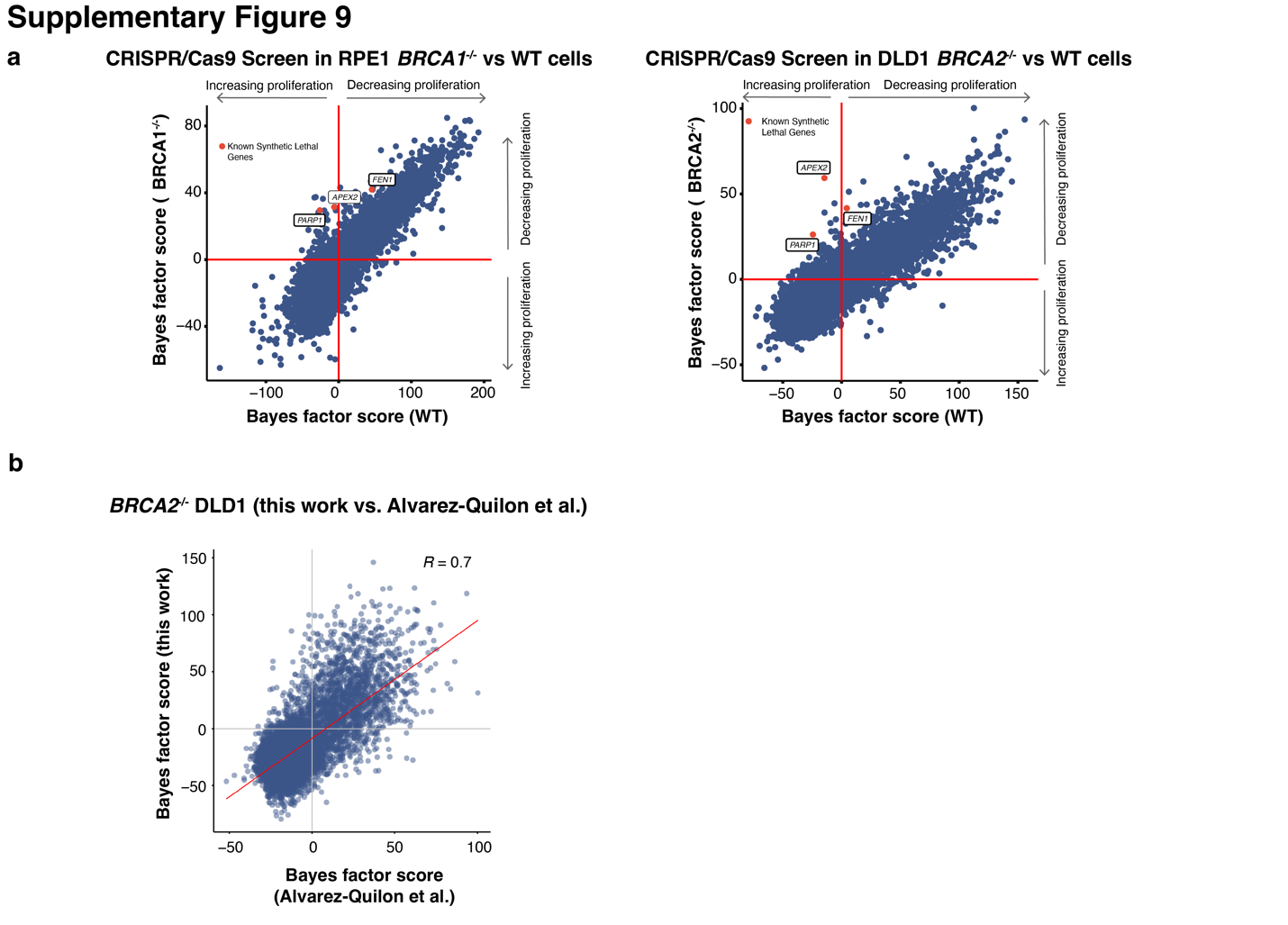


**Fig S10. Bayes factor scores of genome-wide CRISPR/Cas9 screen. (a)** A scatterplot of gene BF scores for *BRCA1*^-/-^ vs. WT cells and *BRCA2*^-/-^ vs. WT cells. Known synthetic lethal genes for *BRCA1*^-/-^ cells and *BRCA2*^-/-^ cells are highlighted. **(b)** Correlation of CRISPR screen performed in this work and previously published dataset. Both are *BRCA2*^-/-^ DLD1 cells. *R*: Pearson correlation.

**
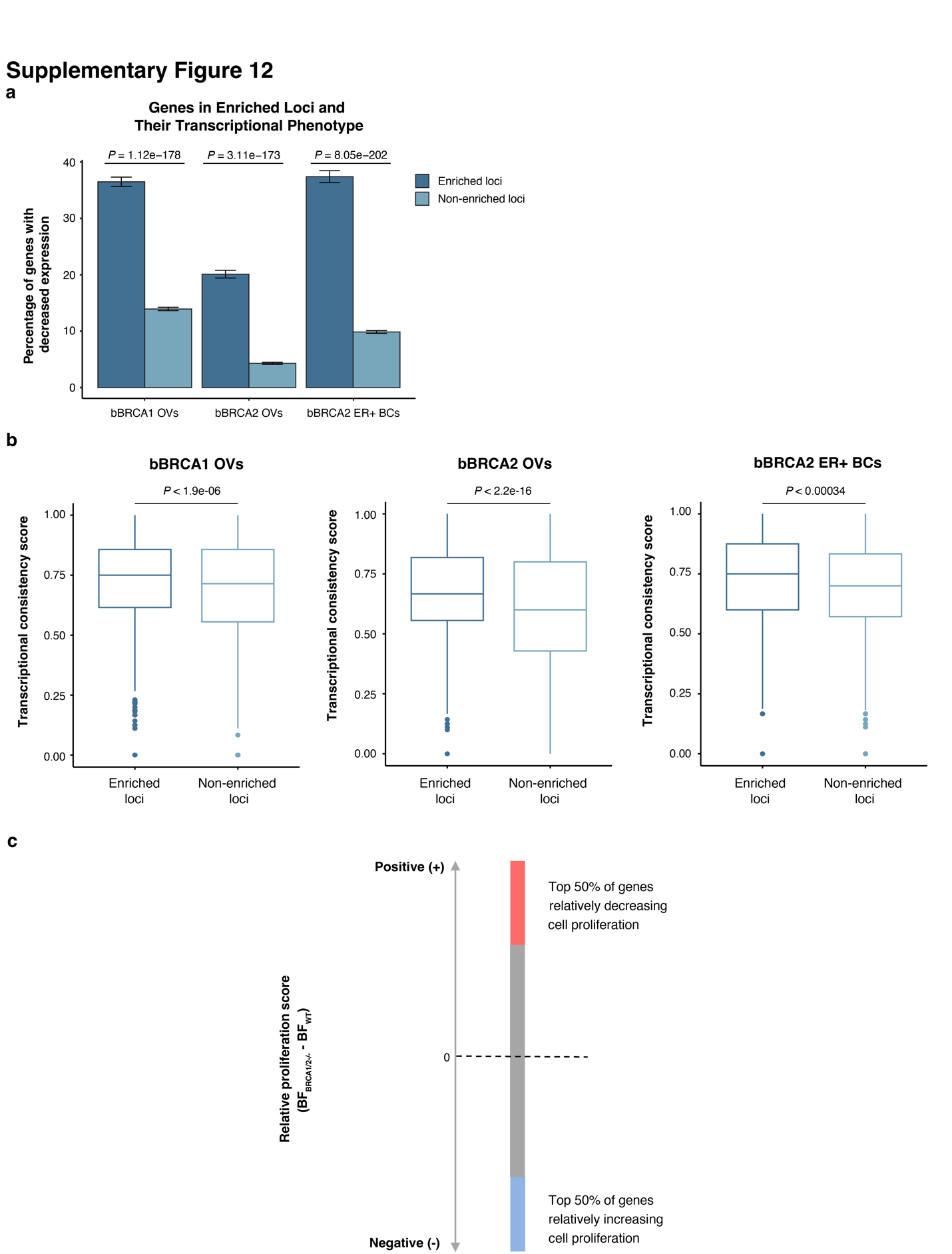
**

**Fig S11. Characterization of transcriptionally decreased genes in enriched loci with deletions in separate bBRCA1 and bBRCA2 tumors. (a)** Genes in the enriched loci with deletions and their transcriptional phenotype. The frequency of transcriptionally decreased genes was compared between genes in the enriched and non-enriched loci. *P*-values were obtained using Fisher’s exact test. Error bars represent standard errors estimated using a proportion test. **(b)** A comparison of TCSs in copy number deletion tumors between genes in the enriched loci and the non-enriched loci (two-sided Mann-Whitney U test). The three horizontal lines in the boxplot proceed from top to bottom: first quartile, median, and third quartile. **(c)** Identifying genes that relatively increase cell proliferation in the *BRCA1*^-/-^ or *BRCA2*^-/-^ cells. Based on the relative proliferation score (RPS), the top 50% of genes with negative RPS were considered to increase cell proliferation.


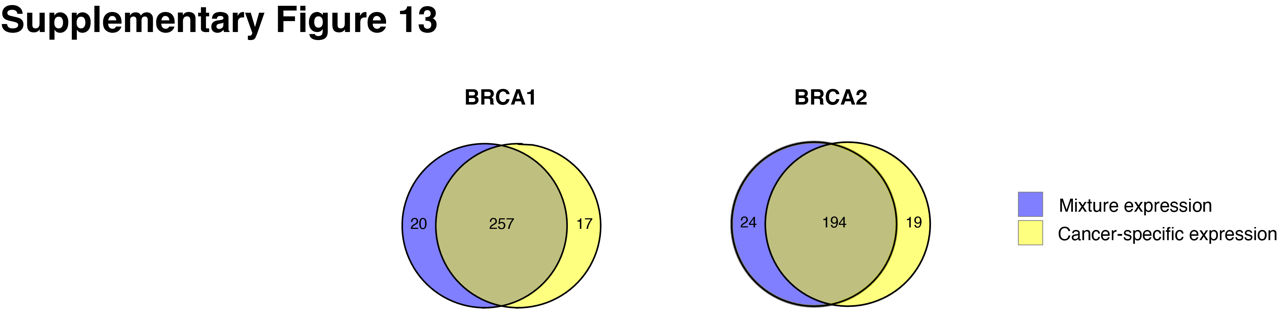


**Fig S12. BRCA1- and BRCA2-related candidates promoting proliferation identified according to cancer-specific expression values imputed by BayesPrism.**


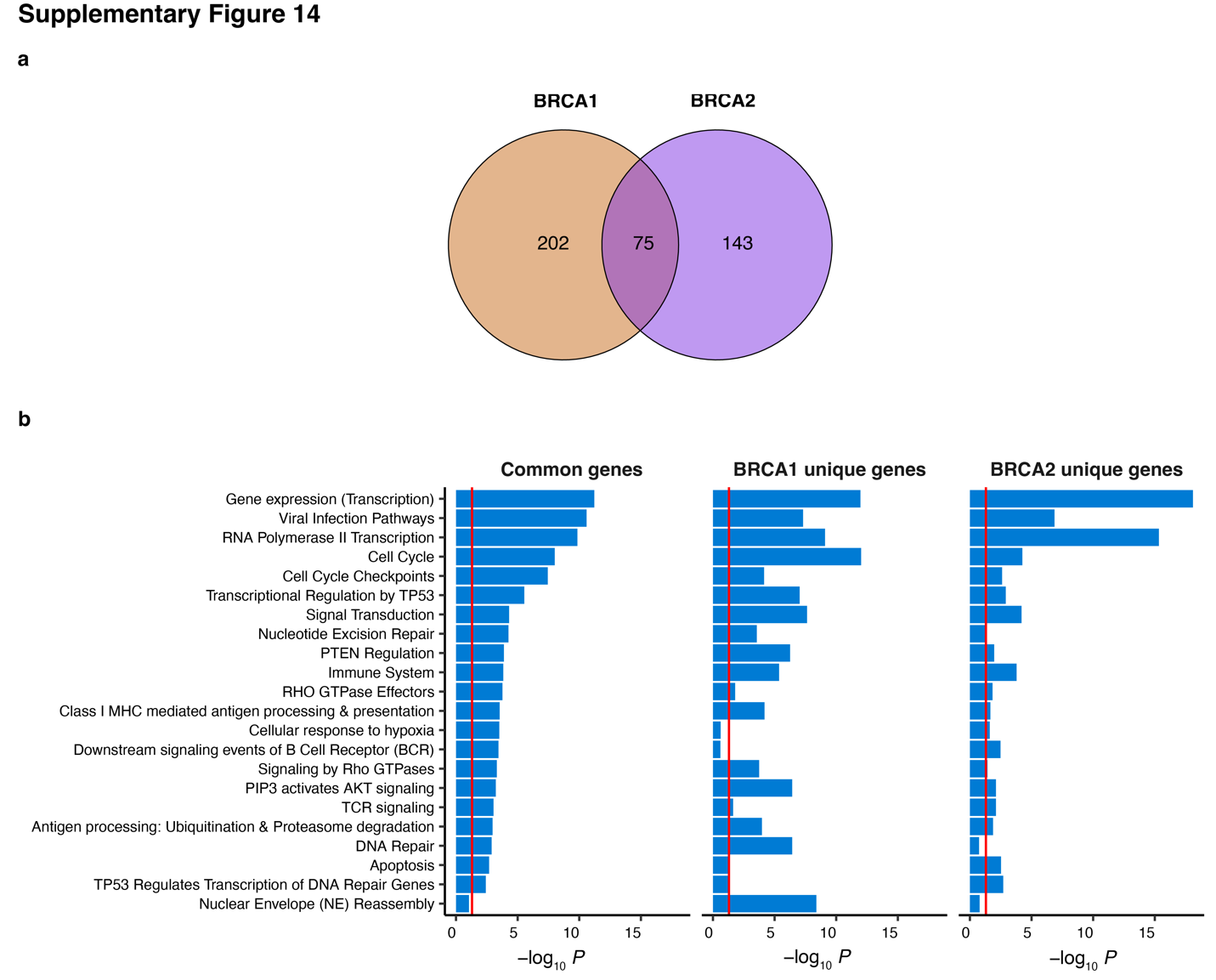


**Fig S13**. **Integrative analysis of BRCA1 and BRCA2. (a)** Intersected genes predicted promoting proliferation in BRCA1- and BRCA2-deficient cells. **(b)** Pathway enrichment in BRCA1- and BRCA2-related genes, which are common between BRCA1 and BRCA2, or BRCA1/BRCA2 unique. The red line indicates *P* value = 0.05.


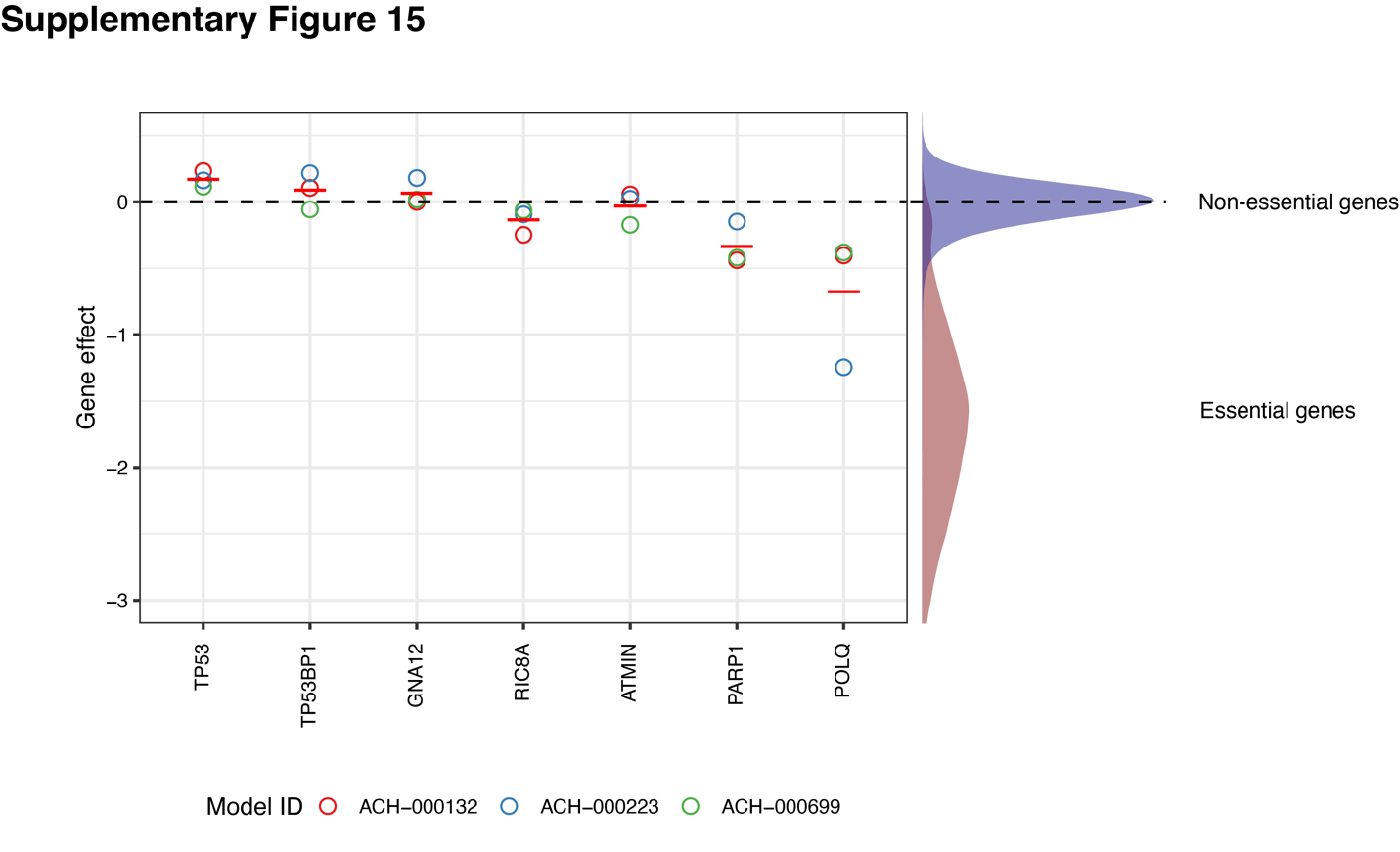


**Fig S14.** **Evaluation of DepMap influence of knockout of various genes in BRCA1 deficient cell lines.** The Y-axis is the gene effect score of cellular proliferation, with higher numbers indicating increased proliferation and lower numbers indicating lower proliferation. The red segment line indicates the mean gene effect score of the 3 cell lines. We consider gene effect values > -0.5 to support increased proliferation in this context.

**
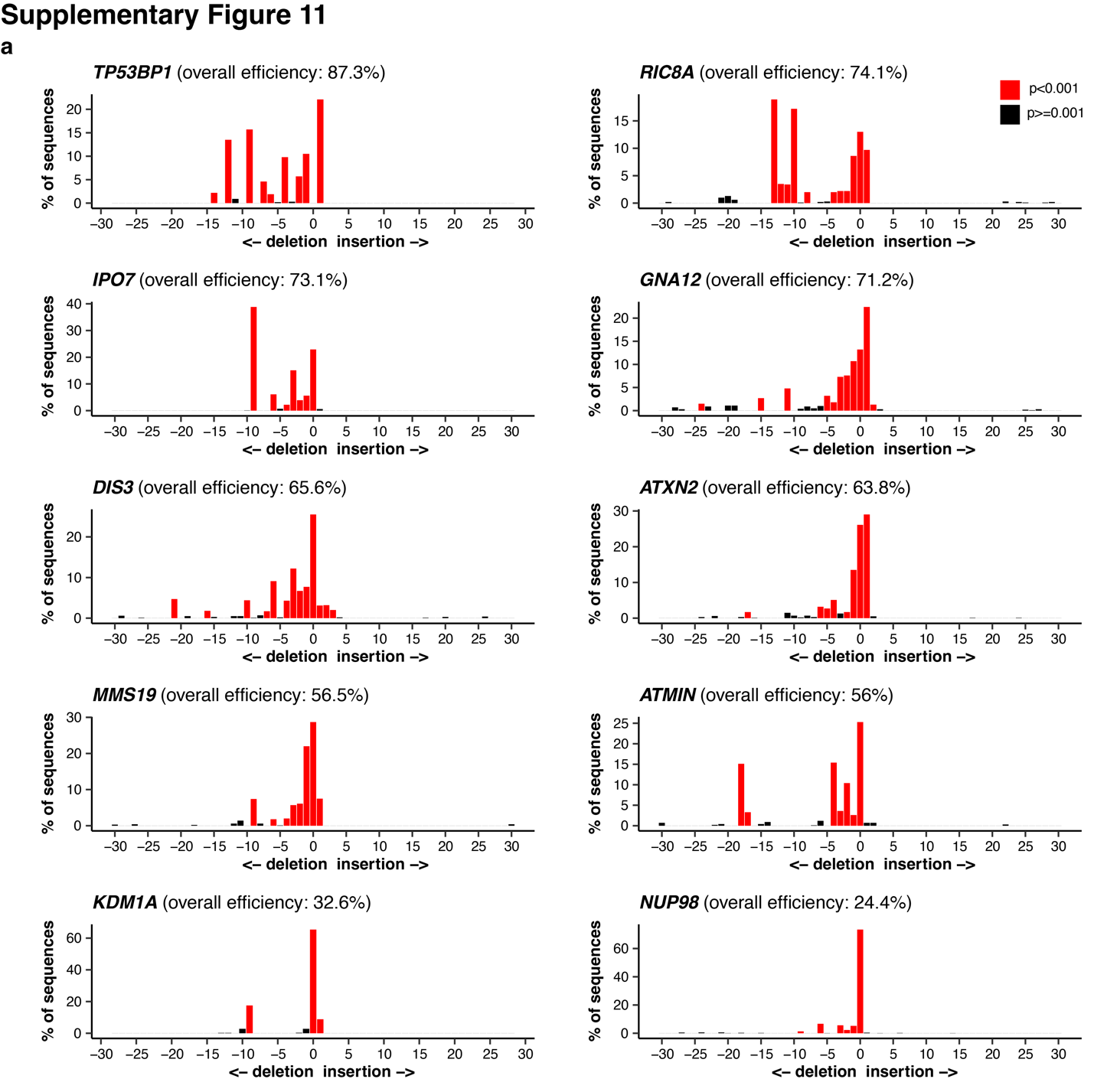
**

**
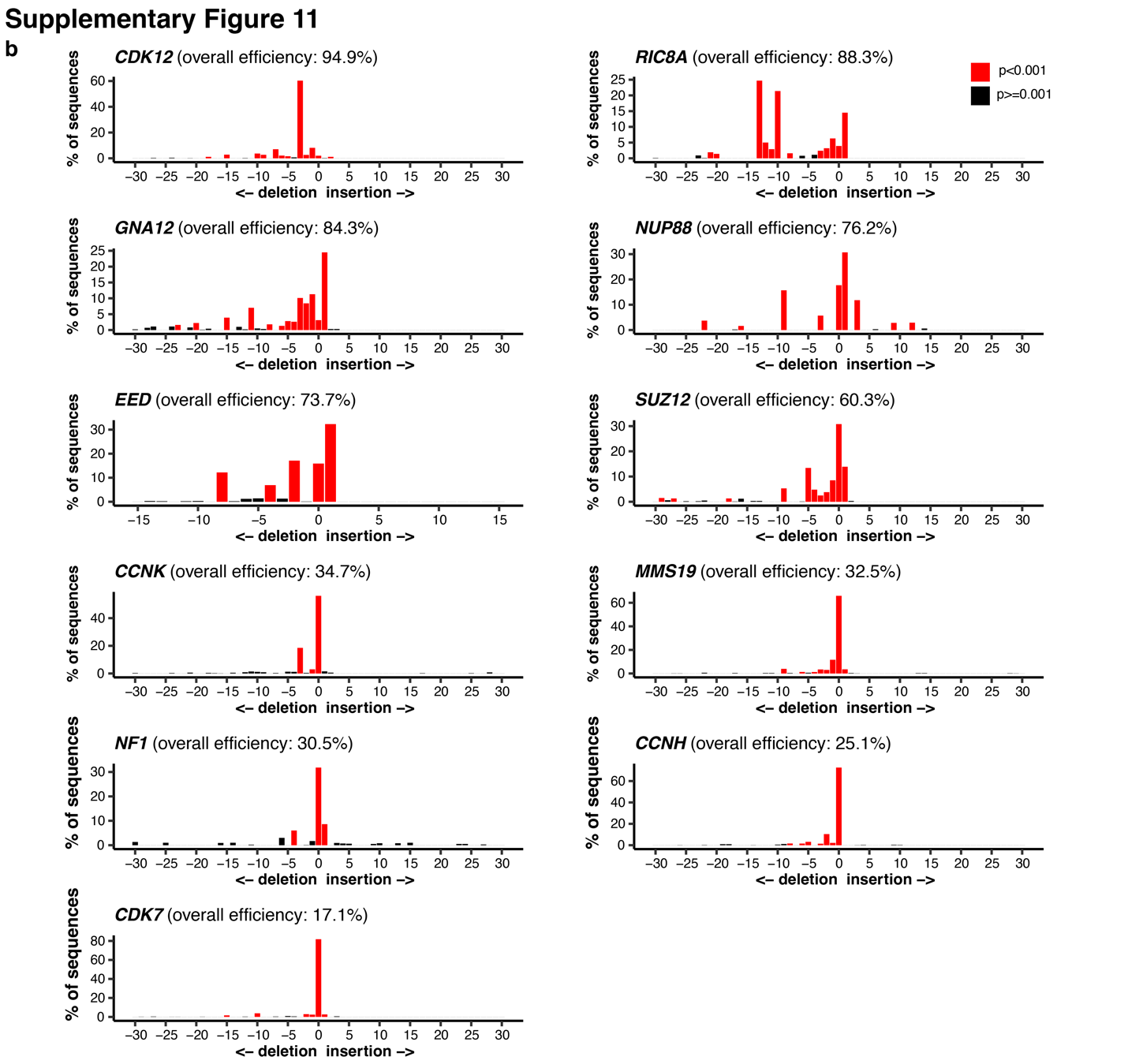
**

**Fig S15. TIDE analysis of CRISPR/Cas9 editing. (a)** TIDE output for the validated genes in *BRCA1*^-/-^ MCF10A cells. **(b)** TIDE output for the validated genes in *BRCA2*^-/-^ MCF10A cells.

**
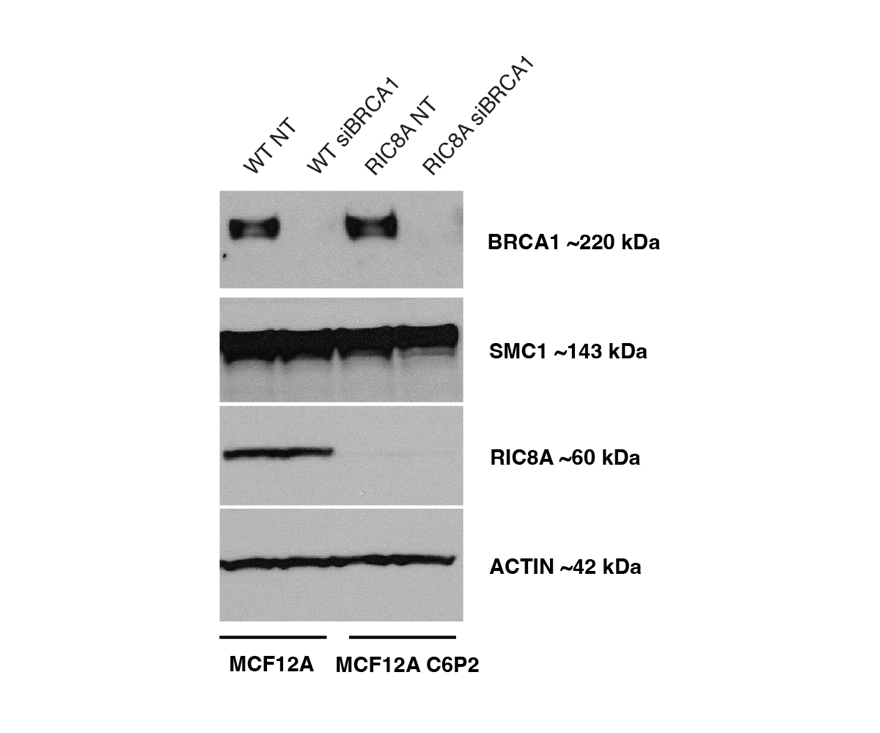
**

**Fig S16. Western blot of RIC8A and BRCA1 in olaparib-treated MCF12A cells. C6P2 were *RIC8A*^-/-^ MCF12A cells.**


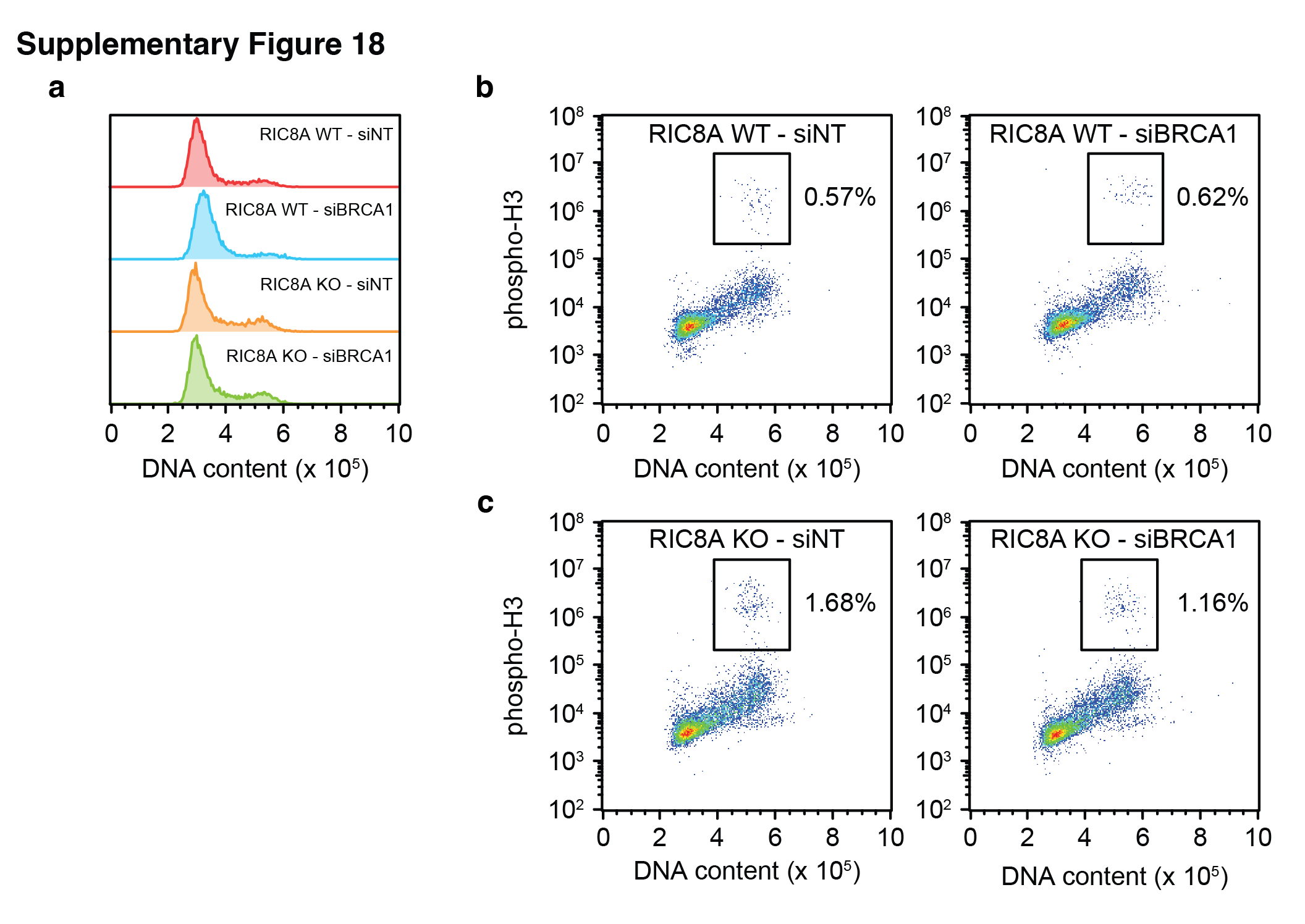


**Fig S17. Fraction of mitotic cells in RIC8A wild-type and knock-out cells treated with BRCA1 siRNA. (a)** DNA content analyzed by flow cytometry of cells with indicated genotype. The top two are RIC8A wildtype (WT), and the bottom two are RIC8A knock-out (KO). Data normalized to the mode. **(b) and (c)** Fraction of mitotic cells inferred by phosphor-H3 positivity in RIC8A WT and KO cells treated with indicated siRNA, respectively.
